# Mutations in FLS2 Ser-938 Dissect Signaling Activation in FLS2-Mediated Arabidopsis Immunity

**DOI:** 10.1371/journal.ppat.1003313

**Published:** 2013-04-18

**Authors:** Yangrong Cao, David J. Aceti, Grzegorz Sabat, Junqi Song, Shin-ichi Makino, Brian G. Fox, Andrew F. Bent

**Affiliations:** 1 Department of Plant Pathology, University of Wisconsin-Madison, Madison, Wisconsin, United States of America; 2 Department of Biochemistry and Center for Eukaryotic Structural Genomics, University of Wisconsin-Madison, Madison, Wisconsin, United States of America; The University of North Carolina at Chapel Hill, United States of America

## Abstract

FLAGELLIN-SENSING 2 (FLS2) is a leucine-rich repeat/transmembrane domain/protein kinase (LRR-RLK) that is the plant receptor for bacterial flagellin or the flagellin-derived flg22 peptide. Previous work has shown that after flg22 binding, FLS2 releases BIK1 kinase and homologs and associates with BAK1 kinase, and that FLS2 kinase activity is critical for FLS2 function. However, the detailed mechanisms for activation of FLS2 signaling remain unclear. The present study initially identified multiple FLS2 in vitro phosphorylation sites and found that Serine-938 is important for FLS2 function in vivo. FLS2-mediated immune responses are abolished in transgenic plants expressing *FLS2_S938A_*, while the acidic phosphomimic mutants FLS2_S938D_ and FLS2_S938E_ conferred responses similar to wild-type FLS2. FLS2-BAK1 association and FLS2-BIK1 disassociation after flg22 exposure still occur with FLS2_S938A_, demonstrating that flg22-induced BIK1 release and BAK1 binding are not sufficient for FLS2 activity, and that Ser-938 controls other aspects of FLS2 activity. Purified BIK1 still phosphorylated purified FLS2_S938A_ and FLS2_S938D_ mutant kinase domains in vitro. Phosphorylation of BIK1 and homologs after flg22 exposure was disrupted in transgenic *Arabidopsis thaliana* plants expressing *FLS2_S938A_* or *FLS2_D997A_* (a kinase catalytic site mutant), but was normally induced in FLS2_S938D_ plants. BIK1 association with FLS2 required a kinase-active FLS2, but FLS2-BAK1 association did not. Hence FLS2-BIK1 dissociation and FLS2-BAK1 association are not sufficient for FLS2-mediated defense activation, but the proposed FLS2 phosphorylation site Ser-938 and FLS2 kinase activity are needed both for overall defense activation and for appropriate flg22-stimulated phosphorylation of BIK1 and homologs.

## Author Summary

As a Pattern-Recognition Receptor (PRR) that detects conserved microbial molecules, FLS2 mediates the plant innate immunity responses triggered by bacterial flagellin or the flagellin epitope flg22. Even though several protein kinases including BAK1, BKK1, BIK1 are known to play early roles in FLS2-mediated signaling, the specific FLS2 phosphorylation and kinase activities during receptor activation after ligand binding remained unclear. We used in vitro and in vivo methods to identify Ser-938 as a potential phosphorylation site of FLS2. Immune responses are blocked when Ser-938 is mutated to alanine but remain normal when mutated to the phosphorylation-mimic residues aspartate or glutamate, suggesting that phosphorylation of Ser-938 is critical for defense activation after flagellin is detected. Phosphorylation of BIK1 and BIK1-homologous proteins is dependent on FLS2 Ser-938, but the widely noted release of BIK1 from FLS2 and binding of BAK1 to FLS2 after flg22 exposure proceed even when Ser-938 is mutated and defense signaling is blocked, suggesting that phosphorylation of Ser-938 and BIK1 phosphorylation prior to release are key steps controlling defense activation after the FLS2 receptor initially detects flagellin.

## Introduction

Pattern-Recognition Receptors (PRRs) can initiate innate immunity by perception of conserved Pathogen- (or Microbe-) Associated Molecular Patterns (PAMPs or MAMPs) [1], [2]. This process, which has been termed PAMP-triggered immunity (PTI), serves as an initial defense response against pathogens. *Arabidopsis thaliana* FLAGELLIN-SENSING 2 (FLS2) [3]–[5] and EF-Tu RECEPTOR (EFR) [6], which detect bacterial flagellin and elongation factor-Tu, respectively, are particularly well-studied examples of plant PRRs along with rice Xa21, which recognizes a sulfated peptide made by some *Xanthomonas* pathogens [7], [8]. FLS2, EFR, and Xa21, as well as other plant PRRs and many animal Toll-like receptors (TLR), belong to the non-RD receptor-like kinase (RLK) family [9]. Named due to the absence of the otherwise widely conserved arginine (R) residue adjacent to the catalytic aspartate (D) in the activation loop, non-RD kinases differ from the widespread RD kinases in that at least some non-RD kinases do not autophosphorylate residues located in the activation loop to facilitate phospho-transfer activity [10], [11]. Hence the phosphorylation of these PRR non-RD kinases is of particular interest for further study.

Prior to ligand exposure, FLS2 is present in FLS2-FLS2 and FLS2-BIK1 complexes that are detectable by co-immunoprecipitation [12]–[14]. FLS2 also associates with close homologs of BIK1 such as PBS1, PBL1 and PBL2, all of which are predicted cytoplasmic protein kinases [13]. BIK1 is phosphorylated and released from FLS2 complexes after cellular exposure to flg22 (a synthetic 22-amino acid peptide based on the recognized epitope of flagellin) [13], [14]. Upon perception of flg22, FLS2 very rapidly forms a heteromer with the LRR-RLK BAK1 or its homolog BKK1 [5], [15], [16]. Moreover, the cellular level of FLS2 was recently shown to be post-translationally regulated by ubiquitination [17]. The fact that FLS2 is targeted for degradation by the two U-box E3 ubiquitin ligases PUB12 and PUB13 after they are phosphorylated by BAK1 suggests that BAK1 regulates the degradation of FLS2 [17]. During PTI, early responses include production of reactive oxygen species (ROS), MPK phosphorylation, and ion channel activation [18]–[21]. Longer-term PTI responses include callose deposition, ethylene production, seedling growth inhibition, and elevated expression of downstream genes associated with antimicrobial defenses [18]. The kinase-associated protein phosphatase (KAPP) was also shown to impact FLS2 signaling, and to associate with FLS2 in yeast two-hybrid experiments [22]. Despite the above advances, many aspects of FLS2 activation, including the site and functional significance of specific phosphorylation events involving FLS2, remain unclear.

Protein phosphorylation, a reversible switching between phosphorylated and unphosphorylated forms, is a common mechanism by which cell signaling pathways are regulated [23]. Autophosphorylation of protein kinases plays an important role in signal transduction pathways for a wide range of plant processes including hormone responses, development, and responses to biotic and abiotic stresses [24]–[29]. Although they resemble serine/threonine protein kinases, BRI1 and BAK1 were recently found to have both serine/threonine kinase and tyrosine kinase activities, which are critical for brassinosteroid signal transduction [28], [30]–[32]. Among plant PRR kinases, rice Xa21 is known to autophosphorylate at Thr-705, in the juxtamembrane domain [33]. But for other PRRs in plants, very little information about autophosphorylation has been reported. Although FLS2 has a possible phosphorylation site at Thr-867 [34], no direct evidence has shown that Thr-867 is an autophosphorylation site. Early phosphoproteomic screens with Arabidopsis did not reveal FLS2 phosphopeptides [35], [36]. A recent high-throughput screen for the autophosphorylation activity of 759 annotated protein kinases from Arabidopsis did not detect autophosphorylation activity for FLS2 (albeit after in vitro translation of a full-length cDNA, and using an anti-phospho-Ser/Thr antibody that may be prone to context-specific limitations in recognition of phosphorylated sites) [37]. These and other studies have, however, indicated that modern wheat germ cell-free translation systems have minimal endogenous phosphorylation activity [37].

In the present study, mass spectrometry (MS) was used to detect apparent autophosphorylation sites of FLS2 kinase purified from a cell-free translation system. Among these sites, we show that Ser-938 is particularly important. FLS2-mediated immune responses including seedling growth inhibition, ROS burst, callose deposition, and MPK phosphorylation are shut down in Arabidopsis *fls2^−^* mutants expressing FLS2_S938A_ protein, as is the phosphorylation of FLS2, while plants expressing the phosphomimic proteins FLS2_S938D/E_ exhibit defense responses similar to those of wild-type plants. The data suggest that phosphorylation of Ser-938 is critical for FLS2-mediated immune responses. flg22-stimulated FLS2-BIK1 dissociation and FLS2-BAK1 association still occur with FLS2_S938A_ protein, but phosphorylation of BIK1 and homologs is absent, suggesting revisions to mechanistic models of FLS2 function.

## Results

### In vitro-synthesized FLS2 is phosphorylated

**Figure 1:**
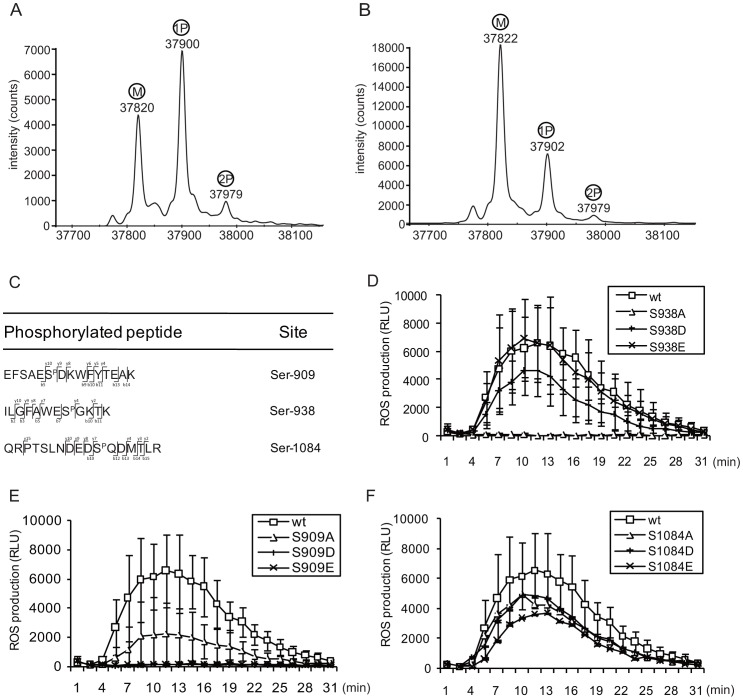
Identification of Ser-938 as a candidate autophosphorylation site of FLS2 *in vitro* and *in vivo*. **A** and **B.** Intact (A) and antarctic phosphatase-treated (B) intracellular domains of FLS2 (aa #840-1172) were analyzed by Mass Spectrometry (MS). M: predicted molecular weight; 1P: predicted peptide with one phosphate group; 2P: predicted peptide with two phosphate groups. **C.** Peptides containing phosphorylated amino acids, identified by mass spectrometry (see also Figure S1). **D–F.** Functional test of three serine sites identified by MS. Reactive oxygen species were measured in leaf discs from transgenic Arabidopsis *fls2-101* plants for 30 min. after treatment with 1 µM flg22. Stable transgenic plants carried *FLS2* serine mutant alleles as specified, with expression driven by native *FLS2* promoter. Data shown are mean ± SE for four to six independent T1 plants per construct. RLU: relative luminescence units; wt: wild-type Col-0 FLS2; S938A: FLS2_S938A_; other *FLS2* alleles similarly labeled.

To characterize the phosphorylation activity of FLS2, the inferred intracellular portion of FLS2 (amino acids 840-end, predominantly a protein kinase domain) was expressed and purified from a wheat germ cell-free expression system and then subjected to MS analysis. A majority of the protein had a mass 80 Da greater than the predicated molecular weight, while a smaller fraction had a mass 159 Da greater than the predicated molecular weight, equivalent to the mass increases expected for the addition of either one or two phosphate groups, respectively (Figure 1A). Although phosphorylation from the expression system is not ruled out, this result is consistent with autophosphorylation activity of the in vitro-synthesized FLS2 kinase. When the purified kinase domain of FLS2 was treated with antarctic phosphatase and analyzed by MS, the abundance of the putative phosphorylated FLS2 peptides was decreased (Figure 1B), a result that is further consistent with the presence of reversible autophosphorylation sites on FLS2.

### Serine-938 is a functionally important FLS2 site

To identify the detected phosphorylation sites, the in vitro-expressed intracellular domain of FLS2 was digested with trypsin and subjected to further mass spectrometry. As shown in Figure 1C and Figure S1, peptide fragments containing phosphorylated Serine 909 (Ser-909), Ser-938 and Ser-1084 were detected with high confidence, suggesting these three serine amino acids as likely in vitro autophosphorylation sites and thus as candidate in vivo autophosphorylation sites.

To allow in vivo studies of protein function the above three serines were then converted by site-directed mutagenesis to phosphorylation-blocking alanine (Ala or A), a substitution that typically does not disrupt overall protein structure [38], or to the carboxylates aspartate (Asp or D) or glutamate Glu or E), whose covalently attached negative charge has been shown in numerous cases to functionally mimic phosphorylated Ser or Thr [39]. These variants of FLS2 were constructed with a C-terminal 3× HA tag, placed downstream of a native *FLS2* promoter, and then transformed into the Arabidopsis *fls2* null mutant line Col-0 *fls2-101*. Wild-type *FLS2-HA* in a similar configuration has previously been shown to function normally [12], [40]. To detect the response to flg22, leaf samples from four to six independent T1 transgenic plants were used for ROS detection after flg22 treatment. Figure 1D and Figure S3E show that for Ser-938, the phosphomimic *FLS2_S938D_* and *FLS2_S938E_* transgenic plants produced an ROS burst comparable to *FLS2_WT_* transgenic Arabidopsis after flg22 treatment, while *FLS2_S938A_* failed to mediate an ROS response to flg22. This suggests that Ser-938 is a functionally significant site on FLS2.

Immunoblot analysis showed that all three mutated Ser-938 variants, including the non-functional FLS2_S938A_, were present in transgenic Arabidopsis (Figure S2A). For wild-type and all three FLS2 variants, further experiments showed presence of the Endoglycosidase H (Endo H)-insensitive form that arises after appropriate processing through the ER and Golgi (Figure S2B; Endo H can cleave mannose rich-oligosaccharides from immature proteins in endoplasmic reticulum (ER) but not the oligosaccharides from mature proteins that have been processed in the Golgi [41]).

Plants expressing *FLS2_S909A_* exhibited a reduced ROS burst, approximately 1/3 the magnitude of the burst mediated by FLS2_WT_ (Figure 1E and Figure S3E). However, plants expressing *FLS2_S909D_* and *FLS2_S909E_* produced little or no ROS after exposure to flg22 (Figure 1E and Figure S3E). These data suggest that integrity of Ser-909 can contribute to FLS2 function, but that Ser-909 phosphorylation apparently does not positively regulate FLS2 activity.

For the site Ser-1084, transgenic Arabidopsis plants expressing *FLS2_S1084A_*, *FLS2_S1084D_* and *FLS2_S1084E_* showed only a minor reduction of ROS production relative to plants expressing a *FLS2_WT_* transgene (Figure 1F and Figure S3E). This suggests that Ser-1084 is relatively unimportant for the tested function of FLS2. For all of the Ser-909, Ser-938 and Ser-1084 mutant plant lines, ROS production was also monitored in the absence of flg22 treatment and only baseline ROS production similar to that of *FLS2_WT_* or *fls2^−^* plants was detected (Figures S3A–C).

A previous study had suggested that Thr-867 in the juxtamembrane domain is a possible autophosphorylation site, because the mutant form FLS2_T867V_ could bind flg22 but lost responsiveness to flg22 treatment [34]. To further examine Thr-867, we constructed *FLS2_T867V_*, *FLS2_T867D_* and *FLS2_WT_* alleles under control of the CaMV *35S* promoter and transformed them into the *Arabidopsis fls2-101* line. Expression of the mutated proteins was confirmed by immunoblot analysis, and responsiveness to flg22 was tested using the seedling growth inhibition assay (Figure S4). Plants expressing *FLS2_T867V_* or the phosphorylation-mimic *FLS2_T867D_* lacked responsiveness to flg22, suggesting as for Ser-909 that Thr-867 is important for FLS2 function, but that phosphorylation of Thr-867 is not likely to be a requirement for FLS2 activity.

The location of Ser-938 and other residues on a folded FLS2 intracellular domain was predicted by homology modeling (Figure S5). The SwissModel and 3D-Jury servers produced multiple high-scoring models of the FLS2 protein kinase domain (∼288 contiguous residues out of the 344 total predicted intracellular residues of FLS2). As is often the case with high-scoring homology models, the small regions of uncertain modeling within the ∼288 amino acid FLS2 kinase models were primarily at surface-exposed loop regions that join two well-anchored helices or sheets. These surface-exposed loop regions are predicted to be relatively flexible in the protein kinases with solved crystal structures. Ser-938 is predicted to reside in kinase subdomain IV, which is near the apex of one such loop (Figure S5). Ser-909 is located in subdomain II, while Ser-1084 is located in subdomain IX (Figure S5). The ∼40 residue “juxtamembrane” region of the intracellular portion of FLS2 was not sufficiently similar to available protein structures to allow reliable homology modeling.

Unlike the sites that are conserved across many or all non-RD kinases (such as kinase catalytic site residues), the functionally important Ser-938 residue and nearby residues (FLS2 residues 933–942) are not well conserved among other non-RD kinases in the LRR-RLK subfamily XII [42] (Figure S6). The conclusion that the FLS2 Ser-938 site is not a universal component of non-RD kinases was further confirmed by functionally testing serine residues in the analogous region of EFR. *EFR* sites coding for Ser-777, Ser-778, and Ser-781 were converted to alanine (A) and aspartate (D) by site-directed mutagenesis and transformed into Col-0 *efr^−^* mutant plants under control of the *EFR* native promoter. Multiple independent T1 transgenic plants were tested for *EFR* function using the seedling growth inhibition assay. As shown in Figure S7, these phosphorylation-blocked (EFR_S777A_, EFR_S778A_, and EFR_S781A_) and phosphomimic (EFR_S777D_, EFR_S778D_, and EFR_S781D_) variants still conferred responsiveness to elf18, suggesting that unlike FLS2 Ser-938, these EFR serine residues are not essential for EFR function.

In preliminary in vivo phosphorylation assays carried out using protoplasts, phosphorylated FLS2_S938D_ was observed (Figure S8), suggesting that Ser-938 is not the only phosphorylation site on FLS2.

### Ser-938 affects FLS2-mediated responses

**Figure 2:**
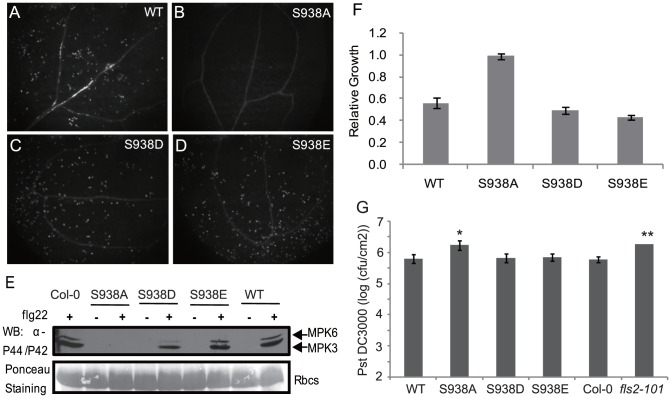
Ser-938 is critical for FLS2-mediated physiological responses. Mutant S938A, S938D, or S938E forms of FLS2 or wild-type FLS2 (WT), as indicated, were expressed under control of *FLS2* promoter in Arabidopsis Col-0 *fls2-101*. Kanamycin/hygromycin-resistant T2 transgenic plants (from T1 plants shown in Figure S2A) were examined after treatment with 1 µM flg22. **A–D.** Representative images of callose production in leaves 24 hr after flg22 treatment. **E.** MPK phosphorylation in seedlings before (−) or 15 min. after (+) flg22 treatment, revealed by immunoblot after SDS-PAGE using the anti-P44/P42 antibody. Lower gel reveals similar loading of total protein. **F.** Response to flg22 in seedling growth inhibition assays. Relative growth is seedling fresh weight after flg22 treatment, divided by average weight of untreated seedlings. **G.** Leaf populations of *P. syringae* pv. *tomato* strain DC3000 3 days after inoculation. Data are mean ±SE for three separate experiments each involving three replicates (total n = 9). * and ** indicate T-test significant difference compared with Col-0 at P<0.05 and P<0.01, respectively.

To further study the role of the FLS2 phosphorylation site Ser-938, transgenic Arabidopsis expressing *FLS2_S938A/D/E_* were tested for characteristic FLS2-dependent plant responses after elicitation with flg22. As shown in Figure 2A–D and Figure S3D, *FLS2_WT_*, *FLS2_S938D_*, and *FLS2_S938E_* transgenic plants accumulated callose after flg22 treatment, but for *FLS2_S938A_* transgenic plants, callose presence did not exceed that of untreated controls. MPK3 and MPK6 became phosphorylated after flg22 exposure of plants expressing *FLS2_WT_*, *FLS2_S938D_*, and *FLS2_S938E_*, but MPK3/6 phosphorylation was absent in *FLS2_S938A_* transgenic Arabidopsis (Figure 2E). A similar loss of flg22-responsiveness was observed in seedling growth inhibition assays (Figure 2F). Lastly, we inoculated different transgenic plants with *Pseudomonas syringae* pv. *tomato* strain DC3000 and found (Figure 2G) that *FLS2_S938A_* transgenic Arabidopsis was more susceptible than Col-0, while *FLS2_S938D/E_* plants were similar to Col-0 plants, demonstrating that availability of FLS2 Ser-938 for phosphorylation or for phosphomimic status is critical in mediating the overall response to *P. syringae* pathogens.

### Association/dissociation of FLS2 with BIK1 and BAK1 is not affected by mutation of FLS2 Ser-938

**Figure 3:**
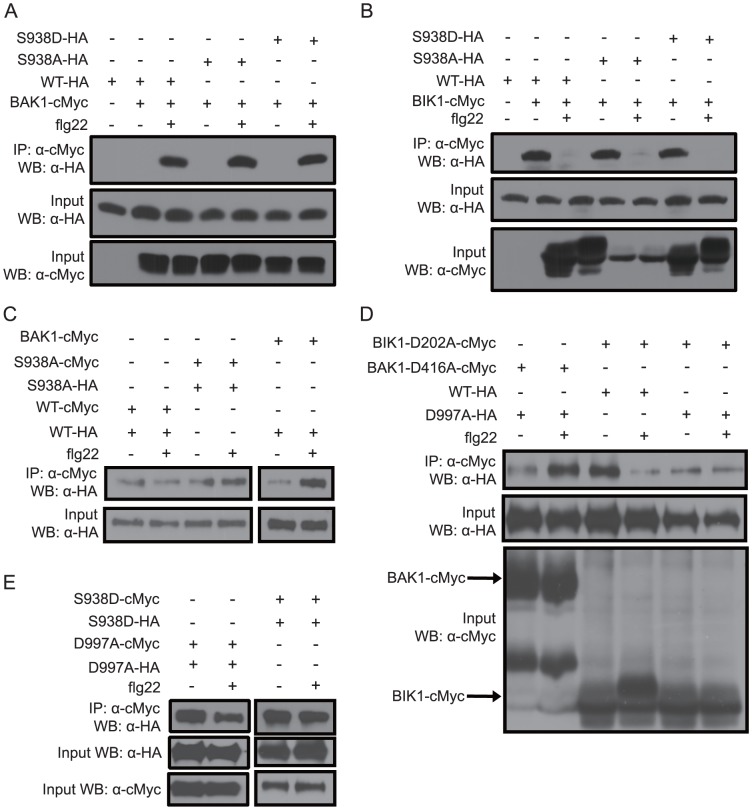
Interaction of BAK1 or BIK1 with variants of FLS2. HA-tagged full-length FLS2 wild type (WT), S938A, S938D or D997A (as labeled), and cMyc-tagged full length BAK1 or BIK1, were coexpressed under control of *35S* promoters in Arabidopsis *fls2-101* protoplasts. Protoplasts were harvested prior to (−) or 15 min. after (+) treatment with 1 µM flg22. Coimmunoprecipitation was carried out using anti-cMyc antibody. Input blots are from SDS-PAGE of total protein extracts; each lane was loaded with equivalent volumes of total protoplasts. WB: antibody used to probe immunoblot. **A.** FLS2_WT_, FLS2_S938A_ and FLS2_S938D_ interact with BAK1 upon flg22 treatment. **B.** BIK1 dissociates from FLS2_WT_, FLS2_S938A_ and FLS2_S938D_ after flg22 treatment. **C.** FLS2-FLS2 association before and after flg22 exposure is not reduced when both FLS2 partners carry the *FLS2_S938A_* mutation. FLS2-BAK1 interaction from the same experiment is shown as a control (all six lanes in C from same protoplast batch, gel, blot and immunodetection). **D.** FLS2_D997A_ interacts with BAK_D416A_ upon flg22 treatment. FLS2_D997A_ does not interact as well as FLS2_WT_ with BIK1_D202A_ before flg22 treatment, and flg22-elicited release of BIK1 is not detected. **E.** FLS2_S938D_, and separately, FLS2_D997A_, form FLS2-FLS2 associations before and after flg22 treatment.

With the knowledge that Ser-938 is a functionally important site of FLS2, we turned to investigate mechanisms that may regulate signal transduction downstream of flagellin or flg22 perception. First, we tested whether the interactions of FLS2 with BIK1 and BAK1 are affected by FLS2 Ser-938. BIK1-cMyc and BAK1-cMyc fusion proteins were transiently expressed together with *FLS2_WT_*, *FLS2_S938A_* or *FLS2_S938D_* in protoplasts made from Arabidopsis *fls2-101* plants, and then coimmunoprecipitation of these FLS2 proteins was monitored after immunoprecipitation using anti-cMyc antibodies. The interactions between FLS2 and BIK1 or BAK1 were not altered when Ser-938 of FLS2 was mutated. As shown in Figure 3A and 3B, the positive control interactions of FLS2_WT_ with BIK1 and BAK1, before and after exposure to flg22, respectively, were as expected from the published literature [5], [13], [14], [43]. BIK1 and BAK1 interaction patterns very similar to those of FLS2_WT_ were also observed with mutated FLS2_S938A_ and FLS2_S938D_. These data indicate not only that Ser-938 is critical for FLS2 activities other than reversible FLS2-BIK1 and FLS2-BAK1 interactions, but also that flg22-elicited FLS2-BIK1 dissociation and FLS2-BAK1 association are not sufficient for FLS2 activity.

### FLS2-FLS2 association is not dependent on Ser-938 or FLS2 kinase activity

Wild-type FLS2 is present in FLS2-FLS2 associations both before and after exposure to flg22 [12]. FLS2_S938A_, FLS2_S938D_, and FLS2_D997A_ also readily formed FLS2-FLS2 associations (Figure 3C and 3E; experiments done in an *fls2-101* genetic background). As with FLS2-BIK1 dissociation and FLS2-BAK1 association, no alteration in FLS2-FLS2 associations was detected when Ser-938 was mutated into a non-phosphorylatable alanine.

### FLS2 kinase activity is required for normal FLS2-BIK1 association, but not for flg22-dependent FLS2-BAK1 association

Kinase-dead versions of FLS2, BIK1 and BAK1 were used to further dissect flg22-dependent protein association/dissociation events. In *fls2-101* cells that carry a wild-type (non-epitope tagged) BAK1 and BIK1 but lack any FLS2 not supplied transgenically, kinase-dead FLS2_D997A_ still associated with kinase-dead BAK1_D416A_ after cells were exposed to flg22 (Figure 3D). Kinase-dead BIK1_D202A_ still associates with wild-type FLS2 and dissociates after cells are exposed to flg22 (Figure 3D). This confirms recent findings [14], [44]. The novel results focus on FLS2 interaction with BIK1 when FLS2 lacks kinase activity. We found that FLS2 kinase activity is required for normal levels of association between FLS2 and kinase-dead BIK1_D202A_ prior to flg22 exposure (Figure 3D).

### In vitro phosphorylation of FLS2 by BIK1 is enhanced by, but does not require, a kinase-active FLS2 or phosphorylation/phosphomimic at FLS2 Ser-938

**Figure 4:**
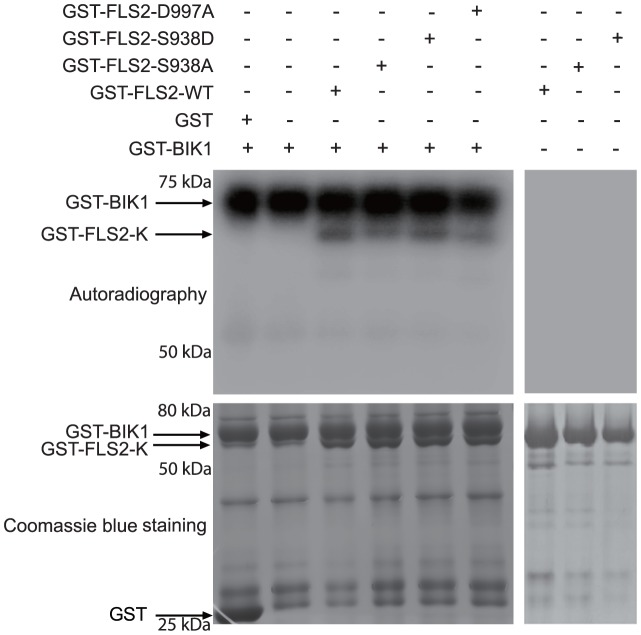
Transphosphorylation of FLS2 kinase domain by BIK1. Kinase domains of FLS2 (wild-type or mutant as specified), and full-length wild-type BIK1 were purified from *E. coli* as GST fusion proteins and used for *in vitro* kinase assays in the presence of [γ ^32^P]-ATP. Upper panel: autoradiograph after SDS-PAGE; lower panel: replicate gel of same samples, stained with Coomassie brilliant blue. Left and right panels are from same experiment but are from two separate gels; gels were exposed together for same period.

BIK1 and some of its homologs were recently shown to contribute to PTI, and to interact with FLS2 when flg22 is absent, and to be released when flg22 is present [13], [14]. The interaction between BIK1 and FLS2 is independent of the protein kinase activity of BIK1 [14]. To test whether the phosphorylation of FLS2 by BIK1 is dependent on FLS2 phosphorylation activity, we carried out in vitro inter-protein phosphorylation assays with BIK1 and different variants of FLS2. We used FLS2_D997A_ as a kinase-dead version of FLS2 that fails to carry out defense signaling [12], [44]; homology models indicate that Asp-997 of FLS2 is a conserved catalytic site residue that is essential in the vast majority of eukaryotic protein kinases [44], [45]. Interestingly, BIK1 could phosphorylate all the variants of FLS2 (Figure 4), but the phosphorylation of FLS2_S938A_ and FLS2_D997A_ proteins was reduced while that of FLS2_S938D_ was strong. An analogous experiment with no GST tag on FLS2 is shown in Figure S9, and gave similar results. These experiments show that in vitro, presence of a phosphomimic or phosphorylatable residue at FLS2 Ser-938 or kinase activity of FLS2 are not essential for phosphorylation of FLS2 by BIK1, but they enhance FLS2 phosphorylation by BIK1. The second and third lanes of Figure 4, and the analogous lanes without and with FLS2_WT_ in Figure S9, also show that any phosphorylation of BIK1 by FLS2 was not detectable over the autophosphorylation activity of BIK1 in these experiments. Figure 4 also shows an FLS2 autophosphorylation assay (no BIK1 present), conducted within the same Figure 4 FLS2+BIK1 experiment (performed on the same day and exposed similarly to the left blot of Figure 4). This latter result further documents the very low autophosphorylation activity of FLS2 relative to the phosphorylation activity of BIK1 in these types of experiments ([14], [44], [46]; see also Figure S8).

### Phosphorylation of BIK1 is dependent on FLS2 Ser-938 and the phosphorylation activity of FLS2

**Figure 5:**
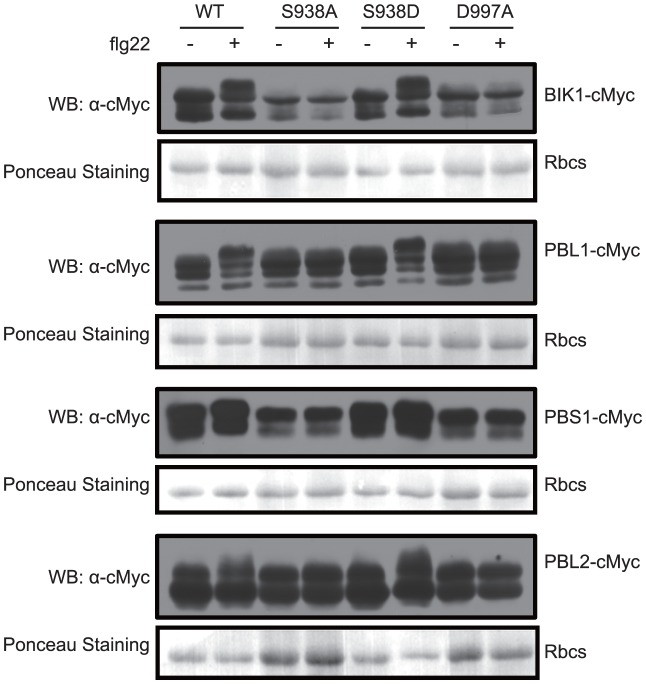
Induced phosphorylation of BIK1 and its homologous proteins under flg22 treatment. cMyc tagged BIK1, PBS1, PBL1, and PBL2 were transiently expressed (under control of 35S promoters) in protoplasts made from Arabidopsis *fls2-101* lines stably transgenic for full-length *FLS2_WT_*, *FLS2_S938A_*, *FLS2_S938D_*, and *FLS2*_D997A_ (under control of *FLS2* promoters; Figure S2A). Protoplasts were treated with (+) or without (−) 1 µM flg22 for 15 min prior to harvest. Total protein extracts were separated by SDS-PAGE and immunoblots were probed with anti-cMyc to detect protein size shift attributable to phosphorylation. Lower panel of each pair shows Ponceau S staining of same immunoblot to assess similarity of total protein levels.

Upon flg22 treatment, BIK1 is phosphorylated and released from FLS2 complexes [13], [14]. This phosphorylation of BIK1 is FLS2-dependent [13], [14], and we showed (above) that the phosphorylation of FLS2 by BIK1 is enhanced by, but does not require, Ser-938 and the kinase activity of FLS2. However, it remained to be shown if the FLS2 Ser-938 site or the kinase activity of FLS2 impacts the phosphorylation status of BIK1 or its partially similar homologs. To address this question, *BIK1* and the genes for the BIK1 homologs *PBS1*, *PBL1*, and *PBL2* were amplified from Arabidopsis, cloned downstream of the CaMV *35S* promoter to encode C-terminal cMyc fusion proteins, and then transiently expressed in Arabidopsis protoplasts made from different transgenic plants. As previously demonstrated for these proteins [13], [14], separation by SDS-PAGE was used to detect altered phosphorylation states as an upward shift in protein mobility. Figure 5 shows that the flg22-elicited phosphorylation of BIK1, PBS1, PBL1, and PBL2 was blocked in *FLS2_S938A_* and *FLS2_D997A_* transgenic Arabidopsis. In the presence of FLS2_WT_, the flg22-elicited phosphorylation levels of PBS1 and PBL2 were lower than those of BIK1 and PBL1, making inferences about PBS1 and PBL2 less certain. However, flg22-elicited phosphorylation of BIK1 and PBL1, and probably also PBS1 and PBL2, was dependent not only on the protein kinase activity of FLS2, but also on the availability of the proposed phosphorylation site Ser-938 or a Ser-938 phosphomimic of FLS2.

## Discussion

The present study sought to identify FLS2 phosphorylation sites and their roles in FLS2-mediated defense signaling. The project was initiated when purified FLS2 obtained from cell-free (in vitro) translation was suggested to have autophosphorylating protein kinase activity. MS analysis identified Ser-909, Ser-938 and Ser-1084 as in vitro autophosphorylation sites or sites phosphorylatable by the wheat germ in vitro translation extract, in either case making them candidate in vivo autophosphorylation sites. Published phosphoproteomic screens of plant extracts have not detected phosphorylation of FLS2 [35]–[37], and our efforts to use MS to detect phosphorylation of FLS2 immunoprecipitated from plant extracts also were not successful (not shown). However, the finding that S938A severely disrupts FLS2-mediated defense activation in vivo without disrupting other FLS2-mediated processes, while FLS2_S938D_ and FLS2_S938E_ retain defense-activating function, strongly implicated Ser-938 as a probable phosphorylation site of substantial functional importance.

The small ‘non-RD kinase’ sub-group of protein kinases was identified based on their lack of the highly conserved Arg-Asp (R-D) residue pair in kinase subdomain VIb [9]. Interestingly, almost all plant and animal immune system PRRs characterized to date are non-RD kinases, suggesting that use of this clade in immune responses became established prior to the divergence of plants and metazoans [9]. Most RD kinases need to autophosphorylate residues located in this activation loop immediately adjacent to the enzyme active site in order to facilitate the phosphotransfer activity of the kinase [47]. For example, Arabidopsis RD kinases BRI1 and BAK1 contain demonstrated autophosphorylation sites in the activation loop (Thr-1049 and either Ser-1044 or Thr-1045 of BRI1 [30]; Thr-446, Thr-449, Thr-450, and Thr-455 of BAK1 [29], [32], [48]). Among predicted Arabidopsis LRR-RLKs, 99% of the RD kinases have a Ser or Thr at the position aligned with BRI1 Thr-1049, compared with 30% for the non-RD kinases [30]. Apparently, this activation loop autophosphorylation site is crucial to the function of RD kinases while it is dispensable for many non-RD kinases, raising questions about the location of important phosphorylation sites on non-RD kinases. The functionally important candidate phosphorylation sites of two non-RD kinases, Thr-705 of Xa21 [33] and Ser-938 of FLS2 (this study), are not located in the kinase activation loop.

Some potential FLS2 autophosphorylation or phosphorylation sites received lower priority in the present study, but merit brief discussion. Arabidopsis FLS2 Thr-867 was previously reported as a potential autophosphorylation site [34], and its location is equivalent to the rice Xa21 autophosphorylation site at Thr-705 [33]. Our data show that *fls2-101* Arabidopsis plants transgenically expressing a phosphomimic FLS2_T867D_ protein or the previously studied non-phosphorylatable FLS2_T867V_ both lose flg22-responsiveness. We also did not detect in vitro autophosphorylation of Thr-867 in mass spectrometry experiments. There are several possible explanations for these results, including: (i) Thr-867 is not a phosphorylation site, but mutations to Asp or Val cause function-blocking disruptions to FLS2 structure; (ii) Thr-867 is not an autophosphorylation site but it is a phosphorylation site, and both phosphorylated and non-phosphorylated status are necessary at some juncture for FLS2 signaling; (iii) Thr-867 is a phosphorylation site but only in vivo, and for this site the T867D mutation does not correctly mimic phosphorylation, or (iv) Thr-867 is a phosphorylation site but only in vivo, and both phosphorylated and non-phosphorylated status are necessary at some juncture for FLS2 signaling. Similar logic may apply to Ser-909 and Ser-1084, the other candidate autophosphorylation sites identified and tested in this study. Integrity of Ser-909 is needed for full FLS2 signaling, but the loss of function of S909D and S909E phosphomimic proteins suggests that phosphorylation of this residue is not needed to activate FLS2 function. Neither phosphorylation nor lack of phosphomimic status at Ser-1084 is required for substantial signaling by FLS2.

It is well established in mammalian systems that autophosphorylation of protein kinase-containing receptors plays a critical role in regulating signal transduction to downstream components after perception of ligand [49], [50]. After discovering that FLS2 Ser-938 is an important candidate phosphorylation site, a number of additional insights into FLS2 function were obtained. If Ser-938 of FLS2 is non-phosphorylatable (FLS2_S938A_), flg22 induction of downstream responses including seedling growth inhibition, ROS burst, callose deposition, MPK phosphorylation and restriction of *P. syringae* growth were all blocked. This was not true of FLS2_S938D_ or FLS2_S938E_. Detectable FLS2 autophosphorylation activity and phosphorylation of BIK1, PBL1, PBS1 and PBL2 were also lost with FLS2_S938A_. However, the ligand-dependent release of BIK1 from FLS2 and the ligand-dependent association of BAK1 with FLS2 were not detectably altered by mutation of Ser-938. As noted in Results, the above yields two important conclusions: (i) Ser-938 controls essential elements of FLS2 activity other than FLS2-BIK1 and FLS2-BAK1 dissociation/association, and (ii) flg22-elicited FLS2-BIK1 dissociation and FLS2-BAK1 association are not sufficient for FLS2 activity.

In vitro, a quantitative contribution of Ser-938 phosphorylation to BIK1 phosphorylation of FLS2 was suggested. However, some phosphorylation of FLS2 by BIK1 still occurred in the absence of FLS2 kinase activity (Figure 3, see FLS2_D997A_), and could occur at sites other than Ser-938 (see FLS2_S938D_ and FLS2_S938A_), and did not strictly require that Ser-938 be phosphorylatable or in the phosphomic state (see FLS2_S938A_). In future studies, it will be of interest not only to directly confirm phosphorylation of Ser-938, but also to learn about the in vivo stoichiometry, timing and target sites of FLS2 phosphorylation by BIK1.

Ser-938 may be a target for autophosphorylation or trans-phosphorylation of FLS2 after flg22 exposure. This too constitutes an interesting topic for future study. However, the findings that S938D or S938E do not constitutively activate FLS2 signaling in the absence of flg22 ligand suggest that phosphorylation of Ser-938 is not sufficient on its own to activate FLS2 signaling.

The tested FLS2 defense signaling functions that were lost due to the FLS2_S938A_ mutation were all retained by FLS2_S938D_ or FLS2_S938E_ phosphomimic proteins. This included flg22-induced phosphorylation of BIK1, PBL1, PBS1 and PBL2 (Figure 5), and the weak in vitro FLS2 autophosphorylation activity (Figure 2A). Although flg22-elicited in vivo phosphorylation of BIK1 is also lost after direct ablation of FLS2 protein kinase activity via the D997A catalytic site mutation (this study), and in plants entirely lacking FLS2 [13], [14], and interactions of FLS2 and kinase-dead BIK1 occurred with wild-type FLS2 but were reduced with a kinase-dead FLS2 (this study), neither we nor others have directly demonstrated phosphorylation of BIK1 by FLS2. Such a demonstration is impeded by inherent biological challenges (high in vitro kinase activity of BIK1 relative to FLS2, including probable BIK1 autophosphorylation activity; presence in vivo of functionally redundant PBS1, PBL1, PBL2 in experiments that might use kinase-dead BIK1). After flg22 stimulation and the postulated FLS2 Ser-938 phosphorylation, BIK1 may be phosphorylated by FLS2, by BIK1 (or homologs), and/or by BAK1. However, the present study did demonstrate that in vivo flg22-dependent phosphorylation of BIK1 and PBL1 (and probably PBS1 and PBL2) requires not only the cellular presence of FLS2 but also a kinase-active FLS2, and an FLS2 with a phosphorylatable or phosphomimic Ser-938.

The above set of findings suggests a chain of events in which FLS2 kinase activity is required for flg22-elicited autophosphorylation or trans-phosphorylation at the proposed Ser-938 phosphorylation site of FLS2, which is a requirement for flg22-induced phosphorylation of BIK1 and its homologs, which is required [13], [14] for FLS2-mediated responses to bacterial flagellin. Although autophosphorylation of FLS2 at Ser-938 by definition would require FLS2 protein kinase activity, that FLS2 protein kinase activity subsequently may be redirected toward other proteins (such as BIK1) after flg22-induced phosphorylation of Ser-938. Alternatively, the change in FLS2 conformation caused by phosphorylation of FLS2 Ser-938 may be sufficient to stimulate BIK1, BAK1 and/or other proteins to activate downstream defense responses, with no further FLS2 kinase activity required.

Ser-938 of FLS2 was not required for release of BIK1, nor was that release sufficient for FLS2-mediated signaling, but it remains to be determined if flagellin/flg22-stimulated release of BIK1 and homologs from FLS2 is required for effective FLS2-mediated signaling. A BIK1-K105E ATP-binding site mutant blocks BIK1 dissociation from FLS2 and exerts a dominant negative impact on PTI [14], but also disrupts BIK1 kinase activity, so although a necessity for release seems likely, it remains unclear if release of BIK1 or homologs from FLS2 is necessary for PTI signaling.

The phosphorylation of BIK1 is induced not only by MAMPs but also by ethylene [13], [14], [51], suggesting that BIK1 plays multiple roles in regulating PTI, ethylene signaling and plant growth [51], [52]. It is interesting that key phosphorylation site mutants of BIK1 and BAK1 can still complement the plant growth defect phenotypes associated with many other types of *bik1* or *bak1* mutations, yet these phosphorylation sites are required for flg22-dependent signaling [14], [32], [53]. This may suggest one of the ways that these proteins achieve specificity as they participate in diverse plant signaling pathways [14].

The present findings also suggest refinements to spatial/temporal models for FLS2 signaling (e.g., [5], [12]–[14], [43]). Autophosphorylation in most cases is not intra-protein self-phosphorylation, but rather, cross-phosphorylation of one protein by a second copy of that protein [54]. Prior to ligand exposure, FLS2 can be found both in FLS2-FLS2 associations and in FLS2-BIK1 (or BIK1 homolog) associations [12]–[14]. If, after flg22 exposure, the protein kinase catalytic site of an FLS2 protein is to be occupied first by the Ser-938 target on a separate FLS2 protein, and then by a BIK1 target, FLS2-FLS2 and FLS2-BIK1 associations would likely be mutually exclusive and involve a swap of interacting partners. For example, FLS2-FLS2 complexes cross-phosphorylate at Ser-938 and dissociate, FLS2-BIK1 associate, quickly followed by BIK1 phosphorylation if FLS2 is phosphorylated at Ser-938, and then BIK1 is released from FLS2 because FLS2 had interacted with flg22 or flagellin. This scheme is somewhat inconsistent with co-IP experimental results, but is consistent with the concepts that equilibrium states generally are not static states (i.e., many of these interactions may have short half-lives within the cell), and is also consistent with the concept that protein-protein interactions that are not sufficiently long-lived to persist throughout co-immunoprecipitation procedures still may be sufficient to foster meaningful signal transduction.

In a separate FLS2/BIK1 model, BIK1-FLS2-FLS2-BIK1 tetramers would be the predominant form prior to ligand exposure. This is more consistent with the available data. In either model, BAK1 apparently participates in the shifts in FLS2/BIK1 interaction, because BAK1 is required for flg22-dependent BIK1 phosphorylation and release from FLS2 [13], and flg22-induced BIK1 phosphorylation requires the FLS2-BAK1 complex [14]. A phosphorylatable FLS2 Ser-938 is not required for flg22-elicited FLS2-BAK1 association, and a kinase-active FLS2 also is not required for flg22-elicited FLS2-BAK1 association, but these FLS2 elements may be required to activate BAK1 for phosphorylation of BIK1 and homologs.

A less likely third model is that the pool of FLS2 that associates with BAK1 is not FLS2 that was bound to BIK1 immediately prior to FLS2-BAK1 association. The FLS2 that interacts with BAK1 may come, for example, from free FLS2 or from FLS2 that resided in FLS2-FLS2 associations that lack BIK1. This model is consistent with previous work suggesting that FLS2-BIK1 dissociation does not need to occur in order for flg22-stimulated FLS2-BAK1 association to occur, but is less consistent with the required role of BAK1 in flg22-dependent BIK1 phosphorylation and release from FLS2 [13], [14].

In yet another model, phosphorylation of Ser-938 might be hypothesized to cause FLS2/BIK1 dimers to be released from FLS2/BIK1-FLS2/BIK1 tetramer complexes, and promote formation of transient FLS2/BIK1/BAK1 complexes that stimulate BIK1 phosphorylation. However, because the detectable levels of FLS2-FLS2 stay constant or increase slightly after flg22 exposure [12], it seems more likely that flg22 does not drive FLS2 dissociation from FLS2. The data are more consistent with a flg22-elicited Ser-938 phosphorylation event altering the FLS2-FLS2 and/or FLS2-BIK1 configuration to activate phosphorylation of BIK1, without dissociation of FLS2 from FLS2.

If larger multi-protein complexes stay temporarily associated after flg22 exposure but prior to BIK1 release (for example, pre-existing FLS2/BIK1-FLS2/BIK1 tetramers remain intact and associate with BAK1 upon flg22 exposure), they would need to exhibit substantial flexibility if one kinase catalytic site is to phosphorylate more than one substrate in that complex. Such flexibility has been proposed for human growth hormone receptors, albeit with only partial supporting data [55]. Alternatively, it is conceptually possible that in a receptor complex carrying proteins in a relatively fixed position, one FLS2 could be oriented to phosphorylate a second FLS2 whose catalytic site is oriented not toward the first FLS2 but instead toward BIK1. However, this type of asymmetric arrangement is not typical of well-described receptor complexes that carry two copies of the same protein [56]–[58]. If the proteins in the receptor complex do stay temporarily bound to each other in relatively fixed positions, it is more likely that they phosphorylate only one partner, supporting a model in which flg22-stimulated FLS2-FLS2 autophosphorylation at Ser-938 does not cause FLS2 to then rotate and phosphorylate BAK1 or BIK1, but instead stimulates BIK1-BIK1 autophosphorylation and/or BAK1 phosphorylation of BIK1.

Spatial models for FLS2 function must also accommodate the fact that the above inter-protein events are physically launched by extracellular interaction of the FLS2 LRR with flagellin or flg22. This FLS2-ligand binding event drives BIK1 release and BAK1 association, and can do so independent of FLS2 Ser-938 and plant defense activation. With each infusion of new experimental data, refined experimental questions arise. In the case of transmembrane LRR-RLK receptors, the demand is increasingly present for methods that can track the activity of single protein molecules over time, or that supply spatially defined protein complex data.

## Materials and Methods

### Cell-free protein synthesis and purification

The standard Center for Eukaryotic Structural Genomics (CESG) platform for cloning [59], protein expression [60], purification [61], and bioinformatics management [62], was utilized to produce FLS2. Briefly, cDNA was cloned into a pEU-His-Flexi vector. Cell-free expression was conducted on two 4-ml scale reactions using WEPRO2240H wheat-germ extract (CellFree Sciences, Yokohama, Japan), unlabeled 20 amino acids, and a Protemist100 protein synthesizer (CellFree Sciences). His-tagged protein was purified by nickel affinity chromatography. The N-terminal His-tag was cleaved with tobacco etch virus protease; and tag-free protein was isolated by subtractive nickel affinity chromatography. Size-exclusion chromatography provided additional purification and permitted exchange of FLS2 into the final buffer (10 mM Bis-Tris, 100 mM sodium chloride, 5 mM dithiothreitol (DTT), 0.02%(w/v) sodium azide, pH 7.0). FLS2 was concentrated to a volume of 150 µl.

### Enzymatic “In Liquid” digestion and nanoLC-MS/MS

“In Liquid” digestion and mass spectrometric analysis was done at the Mass Spectrometry Facility (Biotechnology Center, University of Wisconsin-Madison). In short, 50 µg of purified protein was concentrated via acetone precipitation then re-solubilized under denaturing conditions for tryptic digestion with 10 µl of 8 M urea/100 mM NH_4_HCO_3_, 2.5 µl of 25 mM DTT, 2.5 µl ACN, 32 µl 25 mM NH_4_HCO_3_ and 3 µl trypsin solution (100 ng/µl Trypsin Gold from PROMEGA Corp. in 25 mM NH_4_HCO_3_). Digestion was for 2 hours at 42°C followed by the addition of 3 µl more of trypsin solution (final enzyme: substrate 1∶80) and o/n incubation at 32°C. The reaction was terminated by acidification with 2.5% TFA (Trifluoroacetic Acid) to 0.3% final. 4 µl was loaded on the instrument.

Peptides were analyzed by nanoLC-MS/MS using the Agilent 1100 nanoflow system (Agilent Technologies, Palo Alto, CA) connected to a hybrid linear ion trap-orbitrap mass spectrometer (LTQ-Orbitrap XL, Thermo Fisher Scientific, San Jose, CA) equipped with a nanoelectrospray ion source. Capillary HPLC was performed using an in-house fabricated column with integrated electrospray emitter using 360 µm×75 µm fused silica tubing packed with Jupiter 4 µm C12 particles (Phenomenex Inc., Torrance, CA). Sample loading and desalting were achieved using a trapping column inline with the autosampler (Zorbax 300SB-C18, 5 µM, 5×0.3 mm, Agilent Technologies). HPLC solvents were as follows: for isocratic loading, 1% (v/v) ACN, 0.1% Formic acid; for gradient elution, Buffer A (0.1% formic acid in water) and Buffer B (95% (v/v) acetonitrile, 0.1% formic acid in water). Gradient elution was performed at 200 nL/min and increasing %B from A of 1 to 40% in 135 minutes, 40 to 60% in 20 minutes, and 60 to 100% in 5 minutes. The LTQ-Orbitrap was set to acquire MS/MS spectra in data-dependent mode with MS survey scans from m/z 300 to 2000 collected in centroid mode at a resolving power of 100,000. MS/MS spectra were collected on the 5 most-abundant signals in each survey scan. Dynamic exclusion of 40 seconds intervals with 1.05 m/z above and 0.55 m/z below previously selected precursors was employed. Singly-charged ions and ions for which the charge state could not be assigned were rejected for MS/MS. Raw MS/MS data was compared to a user-defined amino acid sequence database (108,325 protein entries) using an in-house Mascot search engine (Matrix Science, London, UK) with methionine oxidation, asparagine and glutamine deamidation, and serine, tyrosine and threonine phosphorylation as variable modifications. Peptide mass tolerance was set at 20 ppm and fragment mass at 0.6 Da.

### Gene cloning and site-directed mutagenesis

All oligonucleotide primers used for PCR are listed in Table S1. A genomic DNA fragment carrying 1.4 kb directly upstream of the FLS2 translational start site, and the FLS2 coding region up to the stop codon was previously amplified by PCR with *Pfu Ultra* II polymerase and cloned into the pCR8 vector (Invitrogen), resulting in pCR8-Prom-gFLS2 [12]. For site-directed mutagenesis, two complementary primers containing the desired mutation were synthesized, and used to amplify the double-strands plasmid pCR8-Prom-FLS2 by *Pfu Ultra* II polymerase. After digestion with *Dpn* I, PCR products were transformed into *E. Coli* DH5α. The resultant plasmids were verified by sequencing. The wild type and point-mutated versions of FLS2 under its native promoter were recombined into pGWB13 binary vector [63] for further use.

Genomic DNA sequences for *BAK1*, *BIK1*, *PBS1*, *PBL1*, and *PBL2* were amplified by PCR with *Pfu Ultra* II polymerase and inserted into pCR8 vector. The DNA fragment flanking 35S promoter and NOS terminator was amplified from pGWB17 and ligated into pre-linearized plasmid pUC19 by *Sma* I, resulting pUC-GW17 [12]. The genomic DNAs for *BAK1*, *BIK1*, PBS1, *PBL1*, and PBL2 were recombined into pUC-GW17 and verified by sequencing.

### Arabidopsis transformation

All the binary vectors were electroporated into *Agrobacterium tumefaciens* GV3101 (pMP90) and transformed by the floral dip method [64] into the *fls2* T-DNA insertion mutant Col-0 *fls2-101* [65]. Positive transgenic plants were selected on 0.5× MS plate with 25 mg/L kanamycin and 25 mg/L hygromycin after seed surface sterilization by the vapor-phase method [64].

### Seedling growth inhibition and MPK phosphorylation

Transgenic seeds (T1) were screened on 0.5× MS agar plates containing 25 mg/L kanamycin and 25 mg/L hygromycin in a growth chamber for 7 days. Positive transgenic seedlings were transferred into 24-well plates containing liquid 0.5× MS with or without 30 nM flg22 for 10 days of additional growth. Seedling growth inhibition was determined by dividing the weight of flg22-treated seedlings by the mean weight of untreated seedlings from the same experiment. For MPK phosphorylation, T2 transgenic seedlings from 0.5× MS liquid were treated with 2 µM flg22 for 15 minutes, and crude protein was extracted with 2× SDS buffer and separated on 12% SDS-PAGE gel. Phosphorylated MPK3 and MPK6 were detected by anti-P44/P42 antibody (Cell Signaling Technology, Beverly, MA).

### Callose deposition

Callose deposition analysis was performed as described by Adams-Phillips, et al. [66]. In brief, 7-day-old seedlings were treated with 1 µM flg22 in 24-well plates for 24 hours. After fixing overnight in FAA (50% ethanol, 5% glacial acetic acid, 10% formaldehyde solution (initial concentration 37%)), seedlings were stained with aniline blue and callose was visualized using ultraviolet epifluorescence microscopy.

### Oxidative burst

ROS burst experiments performed as described by Sun et al. [12]. In brief, leaf discs were taken from 6-week-old T1 transgenic Arabidopsis and floated on 50 µl 1% DMSO solution in a 96-well plate overnight. 50 µl of aqueous solution carrying 0.5 µl 2 mg/ml luminol in DMSO, 0.5 µl 2 mg/ml horseradish peroxidase (Sigma) and 2 µM flg22 was added just before measurement by plate reader (Centro Xs3 LB 960, Berthold Technology).

### Bacterial growth within leaves

Six-week-old Arabidopsis seedlings were inoculated with *P. syringae* pv. *tomato* strain DC3000 at OD_600_ = 0.0001 in 10 mM MgCl_2_ solution. After 3 days, leaf discs were taken from four inoculated rosette leaves and ground in 10 mM MgCl_2_. The samples were then diluted serially, plated on NYGA plates with 25 mM rifampicin, and colony counts were recorded two days after incubation at 28°C.

### Purification of GST-fusion protein

*FLS2* intracellular domain (encoding amino acids 840–1172) and full length *BIK1* were amplified by PCR and inserted into pGEX 4T-2 at *BamH*I and *Hind*III sites. The resultant plasmids were transformed into BL21 for expression of recombinant protein. BL21 strains harboring plasmid were induced at OD_600_ 0.6 by adding 0.2 mM isopropyl β-D-thiogalactopyranoside (IPTG) at 12°C for 24 hours. The cells were collected at 4°C and resuspended in 10 ml 1× PBS (Sigma) supplemented with 1 mM EDTA, 1 mM phenylmethylsulfonyl fluoride (PMSF), 0.1% Triton X-100, 1 mg/ml lysozyme and put on ice for 30 minutes with slow shaking. After lysis using a French pressure cell (AMINCO, Urbana, IL), the lysate was centrifuged at 14,000 rpm for 30 minutes at 4°C. The resultant supernatant was applied to a column containing glutathione-agarose beads for protein purification. The column was washed with at least 20 bed volumes of 1× PBS. For thrombin digestion to separate FLS2 or BIK1 protein from GST tag, the beads were added to 2 µg/ml thrombin and incubated at 4°C overnight. GST-fusion proteins were eluted with reduced glutathione. GST-free proteins were eluted with 1×PBS. Both GST-fusion proteins and proteins without GST were dialyzed with exchange buffer (50 mM Tris (PH 7.6), 50 mM KCl, 2 mM DTT, and 10% glycerol).

### *In vitro* phosphorylation assays

About 1 µg of purified FLS2 and/or BIK1 intracellular domain as described above were used for in vitro phosphorylation assays. Typically, assays were performed in buffer containing 50 mM Tris (PH 7.6), 50 mM KCl, 2 mM DTT, 5 mM MnCl_2_, 5 mM MgCl_2_, 10 µM ATP and 10 µCi γ-^32^P-ATP at 30°C for 30 minutes. The reaction was stopped by adding EDTA to 1 mM final concentration. Samples were separated on 10% SDS-PAGE gel, and stained with Coomassie blue prior to autoradiography using storage phosphor screens and a Storm 840 phosphorimager (Amersham Biosciences).

### Transient expression in Arabidopsis protoplast

Arabidopsis mesophyll protoplasts were isolated from 6-week-old plants according to the method described by Yoo et al [67]. For coimmunoprecipitation assays, 1 ml of protoplasts (∼10^6^ cells) were transfected with 100 µg plasmid [67], and the transfected protoplasts were incubated at room temperature for about 14 hours. After centrifuge at 500 g for 3 minutes, the protoplasts were frozen in liquid nitrogen and stored in −80°C for use in coimmunoprecipitation assays.

### Coimmunoprecipitation assay

Protoplasts with expression of different proteins as described were lysed with 0.6 ml protein extraction buffer (50 mM Tris (PH 7.6), 150 mM NaCl, 0.5% Triton X-100, and 1× Cocktail protease inhibitor (Sigma)) for 30 minutes on ice. After centrifugation at 14,000 rpm for 15 minutes at 4°C, anti-cMyc was added to the supernatant and incubated at 4°C for 3 hours or overnight, and then 20 µl pre-equilibrated protein A agarose was added for 2 hours with end-to-end shaking at 4°C. Beads were collected by centrifugation at 500 g at 4°C and washed with 1 ml wash buffer (50 mM Tris (PH 7.6), 150 mM NaCl) at least 4 times. After washing, beads were eluted with 50 µl 1× SDS loading buffer by heating at 100°C for 10 min. Eluted proteins were separated on 10% SDS-PAGE gels and transferred to PVDF membrane by electroblotting. Immunoblot detection using the designated horseradish peroxidase-conjugated antibodies was performed using SuperSignal West Pico chemiluminescent substrate (Thermo Scientific, Rockford, IL).

### *In vivo* phosphorylation assay

Protoplasts from *fls2-101* plant transfected with plasmids of FLS2-cMyc, FLS2_S938A_-cMyc, and FLS2_S938D_-cMyc were incubated in W5 solution [67] with 40 µCi/ml inorganic ^32^P-phosphate for 10 hours, and the subjected to immunoprecipitation as above using anti-cMyc. After washing 6 times with 1 ml protein extraction buffer, samples were separated on 10% SDS-PAGE gels and detected by autoradiography using a storage phosphor screen and Storm 840 phosphorimager (Amersham Biosciences).

## Supporting Information

Figure S1 Mass spectrometry analysis of in vitro phosphorylation sites of FLS2.
(PDF)

Figure S2 Expression level of FLS2 in transgenic Arabidopsis.
(PDF)

Figure S3 Oxidative burst under unstimulated conditions.
(PDF)

Figure S4 Thr-867 of FLS2 is not likely to be an autophosphorylation site of FLS2.
(PDF)

Figure S5 Homology model of 3D structure of FLS2 kinase domain, generated using SWISS-MODEL, and annotated amino acid sequence of full-length FLS2 (At5g46330).
(PDF)

Figure S6 Lack of conservation of FLS2 Ser-938 in related non-RD kinases.
(PDF)

Figure S7 Functional test of the response to elf18 peptide mediated by EFR Ser-777, Ser-778, and Ser-781 mutants.
(PDF)

Figure S8 Ser-938 may be important for phosphorylation of FLS2 in vitro and in vivo.
(PDF)

Figure S9 Second example (see also Figure 4) of transphosphorylation of FLS2 kinase domain by BIK1.
(PDF)

Table S1 Primer sequences used in this study.
(PDF)
