# Supplementary material for: Mutations in FLS2 Ser-938 Dissect Signaling Activation in FLS2-Mediated Arabidopsis Immunity
Source: PLoS Pathog. 2013 Apr 18;9(4):e1003313. doi: 10.1371/journal.ppat.1003313 (PMC3630090; doi:10.1371/journal.ppat.1003313)
Supplement: Figure S1 — Mass spectrometry analysis of in vitro phosphorylation sites of FLS2. (PDF) [file ppat.1003313.s001.pdf]

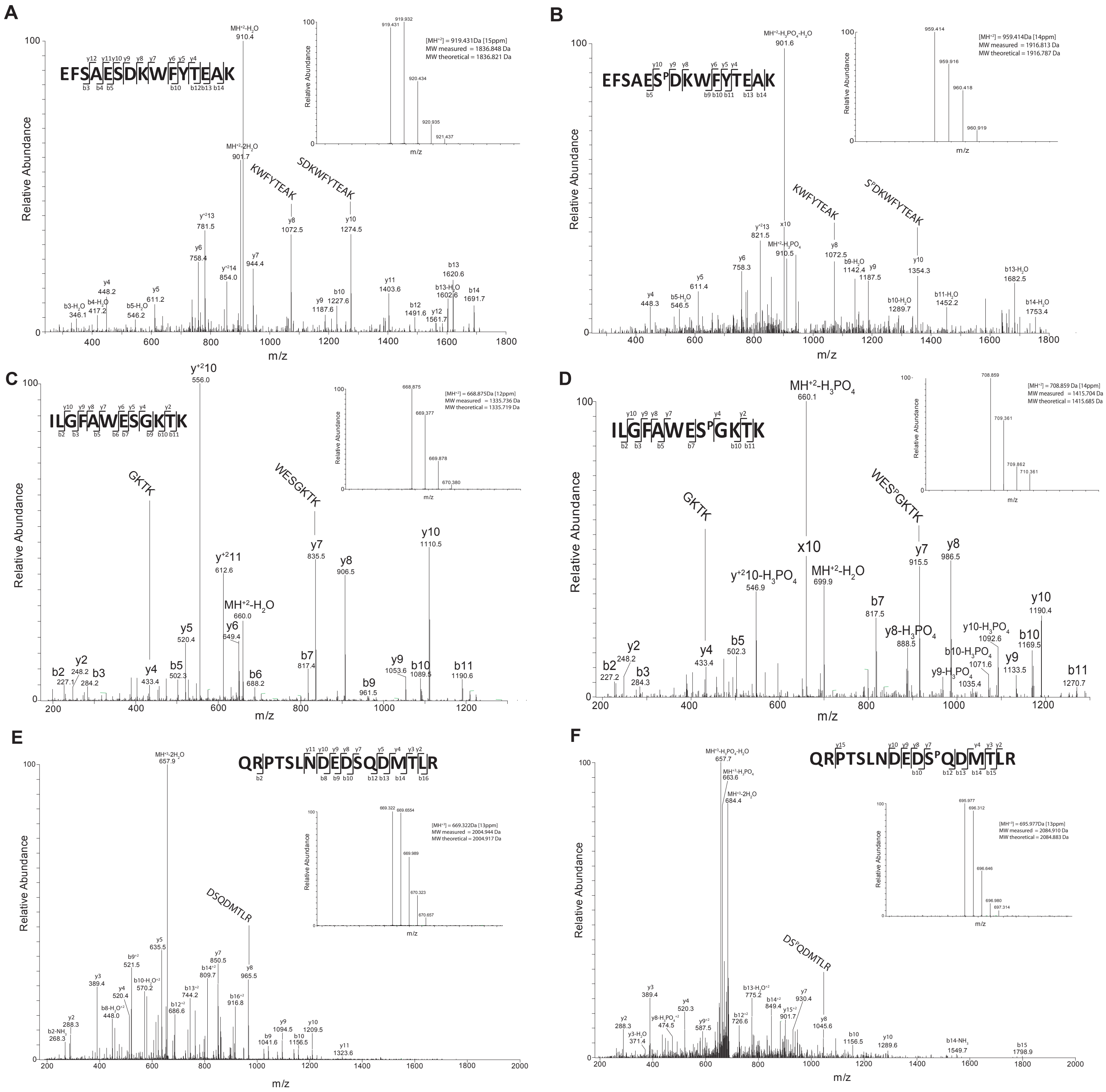

Supplemental Figure 1. Mass spectrometry analysis of *in vitro* phosphorylation sites of FLS2. LTQ-Orbitrap MS/MS spectra identifying FLS2 kinase *in vitro* phosphorylation sites. Each LTQ MS/MS sectrum also contains averaged Orbitrap spectra of its precursor ion with its observed mass and mass error from theoretically determined neutral mass. Both phosphorylated and its unmodified pair are represented.
