# Supplementary material for: Mutations in FLS2 Ser-938 Dissect Signaling Activation in FLS2-Mediated Arabidopsis Immunity
Source: PLoS Pathog. 2013 Apr 18;9(4):e1003313. doi: 10.1371/journal.ppat.1003313 (PMC3630090; doi:10.1371/journal.ppat.1003313)
Supplement: Figure S2 — Expression level of FLS2 in transgenic Arabidopsis. (PDF) [file ppat.1003313.s002.pdf]

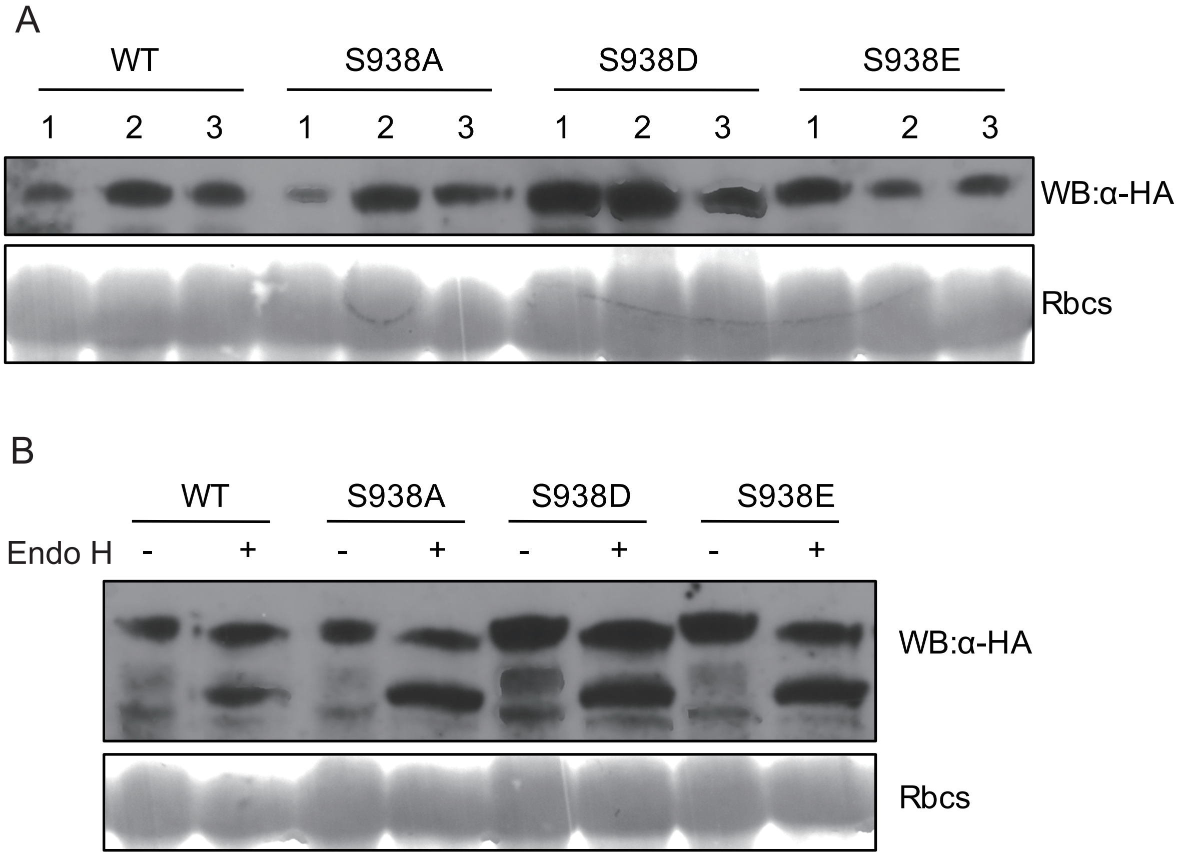

Supplemental Figure 2. Expression level of FLS2 in transgenic Arabidopsis.

A. Expression level of *FLS2* in randomly choosen individual transgenic T1 seedlings from experiment of Figure 1D-F.

B. Impact of FLS2 with mutations at Ser-938 position on FLS2 processing in ER. Western blot shows the results before and after Endo H digestion. WT: FLS2-WT; S938A: FLS2-S938A; S938D: FLS2-S938D; S938E: FLS2-S938E.
