# Supplementary material for: Mutations in FLS2 Ser-938 Dissect Signaling Activation in FLS2-Mediated Arabidopsis Immunity
Source: PLoS Pathog. 2013 Apr 18;9(4):e1003313. doi: 10.1371/journal.ppat.1003313 (PMC3630090; doi:10.1371/journal.ppat.1003313)
Supplement: Figure S3 — Oxidative burst under unstimulated conditions. (PDF) [file ppat.1003313.s003.pdf]

Supplemental Figure 3

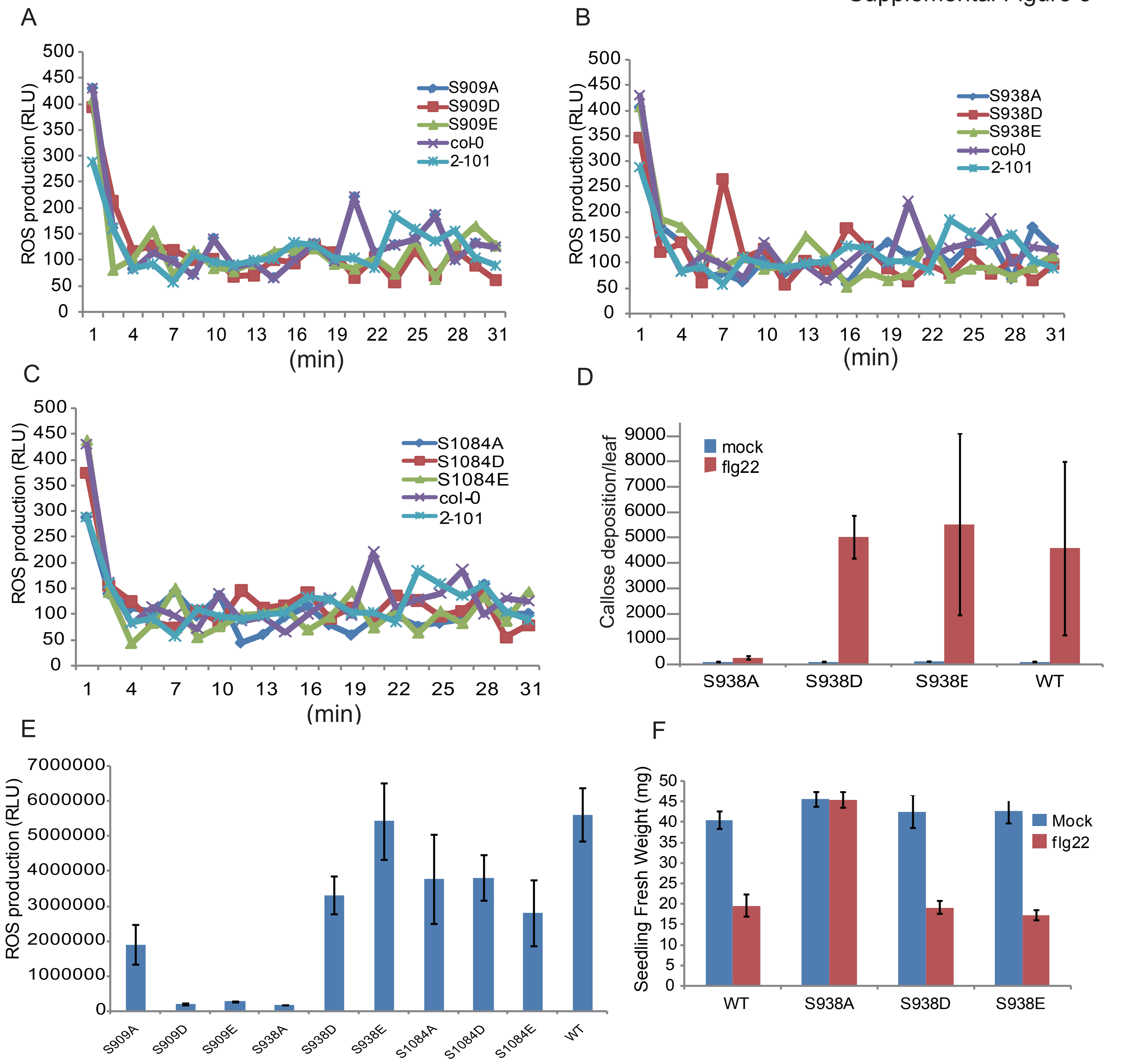

Supplemental Figure 3. Physiological responses under flg22 treatment.

Leaf discs were taken from T2 transgenic Arabidopsis expressing *FLS2-WT*, *FLS2-S909A/D/E* **(A)**, *FLS2-S938A/D/E* **(B)** and *FLS2-S1084A/D/E* **(C)** and measured by luminol assay. Note: Y-axis is magnified about 20-fold compared with Figure 1D, E, and F. **(D)** Quantification of callose depostion as shown in Figure 2A-D by using ImageJ software. Error bar indiactes SD (n=6). **(E)** Quantification of ROS production as shown in Figure 1D-F, each bar indicates area under the curve. Error bar indicates SD (n=8). **(F)** Seedling growth inhibtion assay, same raw data as in Figure 2F, shown as seedling fresh weight.
