# Supplementary material for: Mutations in FLS2 Ser-938 Dissect Signaling Activation in FLS2-Mediated Arabidopsis Immunity
Source: PLoS Pathog. 2013 Apr 18;9(4):e1003313. doi: 10.1371/journal.ppat.1003313 (PMC3630090; doi:10.1371/journal.ppat.1003313)
Supplement: Figure S4 — Thr-867 of FLS2 is not likely to be an autophosphorylation site of FLS2. (PDF) [file ppat.1003313.s004.pdf]

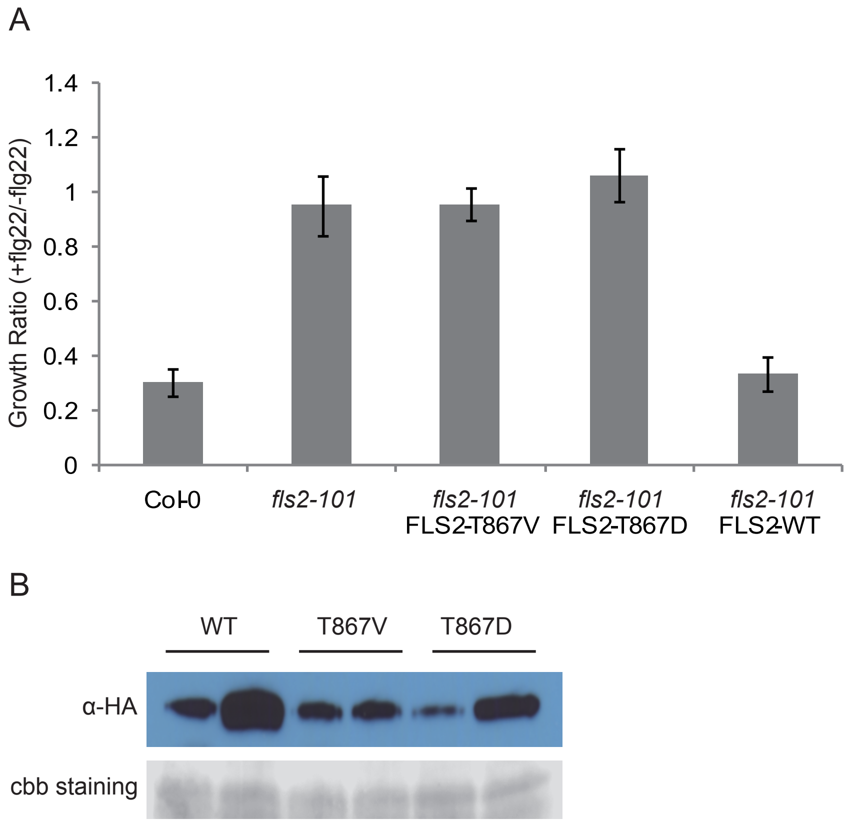

Supplemental Figure 4. Thr-867 of FLS2 is not likely to be an autophosphorylation site of FLS2.

A. Seedling growth inhibition assay was performed by using wild type Col-0 Arabidopsis or Col-0 *fls2-101* transgenic Arabidopsis carrying *FLS2-WT*, *-T867V* and *-T867D*. 7-day-old T1 transgenic seedlings were screened on 1/2 X MS plate containing kanamycin and hygromycin and transferred into liquid 1/2 X MS with or without 1 μM flg22. Seedlings were grown for additional 10 days in the growth chamber. Fresh weight of each seedling was obtained and growth ratio was determined determined by dividing the weight of flg22-treated seedlings by the mean weight of untreated seedlings from the same experiment. Mean ±SE is shown for 6 independent transgenic T1 seedlings.

B. Expression of FLS2 in transgenic lines detected by Western blot with antibody anti-HA. Two T1 transgenic seedlings were randomly selected for this experiment. WT: FLS2-WT; T867V: FLS2-T867V; T867D: FLS2-T867D.
