# Supplementary material for: Mutations in FLS2 Ser-938 Dissect Signaling Activation in FLS2-Mediated Arabidopsis Immunity
Source: PLoS Pathog. 2013 Apr 18;9(4):e1003313. doi: 10.1371/journal.ppat.1003313 (PMC3630090; doi:10.1371/journal.ppat.1003313)
Supplement: Figure S5 — Homology model of 3D structure of FLS2 kinase domain, generated using SWISS-MODEL, and annotated amino acid sequence of full-length FLS2 (At5g46330). (PDF) [file ppat.1003313.s005.pdf]

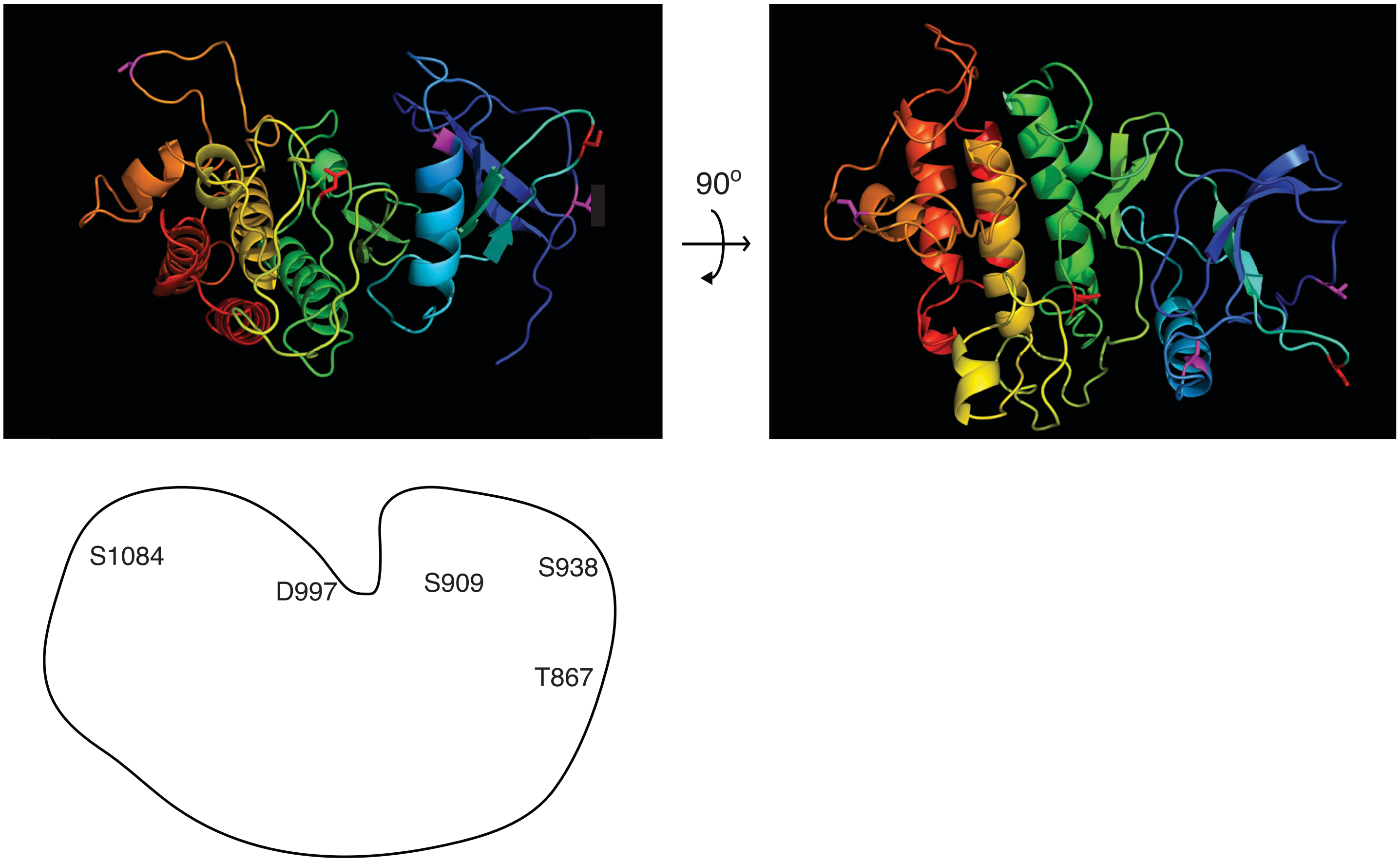

Supplemental Figure 5A. Homology model of 3D structure of FLS2 kinase domain generated using SWISS-MODEL.

FLS2 residues 860-1161 were modelled based on PDB template 2QKW_B (tomato Pto kinase), the top selection by SWISS-MODEL (Arnold, et al. 2006) at the time of the modeling. 3D-Jury results suggested multiple similar-scoring models, including 2QKW-B, 3TL8_A (Arabidopsis BAK1) and 1B6C_B (cytoplasmic domain of Type I TGF-b receptor). Top of model is rotated 90o toward viewer ona horizontal axis in the right image. Rainbow coloring transition is from amino- (blue) to carboxy- (red) termini. Amino acid side chains are represented only for S938 and D997 (red), and T867, S909 and S1084 (purple), which are located in the left image as depictedin the cartoon below that image. Cartoon also emphasizes catalytic cleft of the protein kinase.

Arnold K., Bordoli L., Kopp J., and Schwede T. (2006). The SWISS-MODEL Workspace: A web-based environment for protein structure homology modelling. Bioinformatics, 22,195-201.

1 mkllsktfli ltltffffgi alakqsfepe iealksfkng isndplgvls dwtiigslrh

61 cnwtgitcds tghvvsvsll ekqlegvlsp aianltylqv ldltsnsftg kipaeigklt

121 elnqlilyln yfsgsipsgi welknifyld lrnnllsgdv peeicktssl vligfdynnl

181 tgkipeclgd lvhlqmfvaa gnhltgsipv sigtlanltd ldlsgnqltg kiprdfgnll

241 nlqslvlten llegdipaei gncsslvqle lydnqltgki paelgnlvql qalriyknkl

301 tssipsslfr ltqlthlgls enhlvgpise eigfleslev ltlhsnnftg efpqsitnlr

361 nltvltvgfn nisgelpadl glltnlrnls ahdnlltgpi pssisnctgl klldlshnqm

421 tgeiprgfgr mnltfisigr nhftgeipdd ifncsnletl svadnnltgt lkpligklqk

481 lrilqvsyns ltgpipreig nlkdlnilyl hsngftgrip remsnltllq glrmysndle

541 gpipeemfdm kllsvldlsn nkfsgqipal fsklesltyl slqgnkfngs ipaslkslsl

601 lntfdisdnl ltgtipgell aslknmqlyl nfsnnlltgt ipkelgklem vqeidlsnnl

661 fsgsiprslq acknvftldf sqnnlsghip devfqgmdmi islnlsrnsf sgeipqsfgn

721 mthlvsldls snnltgeipe slanlstlkh lklasnnlkg hvpesgvfkn inasdlmgnt

781 dlcgskkplk pctikqkssh fskrtr**vili ilgsaaalll vlllvlilt**c ckkkekkien

I II

841 ssesslpdld salklkrfep keleqatdsF nsaniigsss lstvykgqlE dgtviavkvl

909 III IV 938 V

901 nlkefsae**s**D kwfyteaktl sqlKhrnlvk ilgfawe**s**gk tKalvlpfme ngnledtihg

VIa VIb VII

961 saapigslLe kidlcvhias gidylhsgyg fPivhcdlkp anilldsdRv ahvsdfgtar

VIII IX

1021 ilgfredgst taStsafegt igylapefay mrkvttKadv fsfgiimmel mtkqrptsln

1084 X XI

1081 ded**s**qdMtlr qlveksigng rkgmvrvldm elgdsivsLk qeeaiedflk lclfctssrp

1141 edrpdmneil thlmklrgka nsfredrned rev

Supplemental Figure 5B. Annotated amino acid sequence of full-length FLS2 (At5g46330). Blue residues indicate trasmembrane domain that separates predicted extracellular N-terminus from predicted intracellular C-terminus. Each kinase subdomain (indicated with Roman numerals) starts with a capitol amino acid letter. Red residues are the three phosphorylation sites identified in vitro, for which amino acid number is also shown.
