# Supplementary material for: Mutations in FLS2 Ser-938 Dissect Signaling Activation in FLS2-Mediated Arabidopsis Immunity
Source: PLoS Pathog. 2013 Apr 18;9(4):e1003313. doi: 10.1371/journal.ppat.1003313 (PMC3630090; doi:10.1371/journal.ppat.1003313)
Supplement: Figure S6 — Lack of conservation of FLS2 Ser-938 in related non-RD kinases. (PDF) [file ppat.1003313.s006.pdf]

```
AT3G47090.1          ----------------------KKRKNNQKINNSAPFTLEIFHEKLSYGDLRNATDGFS
AT3G47580.1          ----------------------RKRRKNQQTNNLVPSKLEIFHEKISYGDLRNATNGFS
AT3G47570.1          ----------------------RKRKKNKETNNPTPSTLEVLHEKISYGDLRNATNGFS
AT3G47110.1          ---------------------------------------------------------FS
AT5G20480.1          ------------------FMKRKKKNNASDGNPSDSTTLGMFHEKVSYEELHSATSRFS
AT5G39390.1          ----------------------KKKN-----------------DKISYEELYNATSGFS
AT2G24130.1          IATPVLCVFGYPLVQRSRFGKNLTVYAKEEVEDEEKQNQDPKYPRISYQQLIAATGGFN
AT5G46330.1 (FLS2)   -------------------KKKEKKIENSSESSLPDLDSALKLKRFEPKELEQATDSFN 871
                                                                       *    .

AT3G47090.1          SSNIVGSGSFGTVFKALLQTENKIVAVKVLN--MQRRGAMKSFMAECESLKDIRHRNLVK
AT3G47580.1          SSNMVGSGSFGTVFKALLPTESKIVAVKVLN--MQRRGAMKSFMAECESLKDTRHRNLVK
AT3G47570.1          SSNMVGSGSFGTVYKALLLTEKKVVAVKVLN--MQRRGAMKSFMAECESLKDIRHRNLVK
AT3G47110.1          SSNLIGSGNFGAVFKGFLGSKNKAVAIKVLN--LCKRGAAKSFIAECEALGGIRHRNLVK
AT5G20480.1          STNLIGSGNFGNVFKGLLGPENKLVAVKVLN--LLKHGATKSFMAECETFKGIRHRNLVK
AT5G39390.1          SSNLIGSGNFSDVFKGLLGLEEKLVAVKVLN--LLKHGATKSFIAECESFKGIRHRNLAK
AT2G24130.1          ASSLIGSGRFGHVYKGVLRN-NTKVAVKVLDP-KTALEFSGSFKRECQILKRTRHRNLIR
AT5G46330.1          SANIIGSSSLSTVYKGQLED-GTVIAVKVLNLKEFSAESDKWFYTEAKTLSQLKHRNLVK 930
                     ::.::**. :. *:*. *    . :*:***:           *  *.: :   :**** :

AT3G47090.1          LLTACASIDFQGNEFRALIYEFMPNGSLDKWLHPEEVEEIHRPSRTLTLLERLNIAIDVA
AT3G47580.1          LLTACASTDFQGNEFRALIYEYLPNGSVDMWLHPEEVEEIRRPPRTLTLLERLNIVIDVA
AT3G47570.1          LLTACSSIDFQGNEFRALIYEFMPNGSLDMWLHPEEVEEIHRPSRTLTLLERLNIAIDVA
AT3G47110.1          LVTICSSSDFEGNDFRALVYEFMPNGNLDMWLHPDEIEETGNPSRTLGLFARLNIAIDVA
AT5G20480.1          LITVCSSLDSEGNDFRALVYEFMPKGSLDMWLQLEDLERVNDHSRSLTPAEKLNIAIDVA
AT5G39390.1          LITVCSSLDSQGNDFRALVYEFMPKGSLDMWLQPEDLESANNHSRSLTFAEKVNIAIDVA
AT2G24130.1          IITTCSKPG-----FNALVLPLMPNGSLERHLYPGEYS-----SKNLDLIQLVNICSDVA
AT5G46330.1          ILGFAWESG----KTKALVLPFMENGNLEDTIHG-------SAAPIGSLLEKIDLCVHIA 979
                     ::  . ..        .**:   : :*.::  :           .         :::  .:*

AT3G47090.1          SVLDYLHVHCHEPIAHCDLKPSNILLDDDLTAHVSDFGLARLLLKFDQESFFNQLSSAG-
AT3G47580.1          SVLDYLHVHCHEPIAHCDLKPSNVLLEDDLTAHVSDFGLARLLLKFDKESFLNQLSSAG-
AT3G47570.1          SVLDYLHVHCHEPIAHCDLKPSNVLLDDDLTAHVSDFGLARLLLKFDEESFFNQLSSAG-
AT3G47110.1          SALVYLHTYCHNPIAHCDIKPSNILLDKDLTAHVSDFGLAQLLLKFDRDTFHIQFSSAG-
AT5G20480.1          SALEYLHVHCHDPVAHCDIKPSNILLDDDLTAHVSDFGLAQLLYKYDRESFLNQFSSAG-
AT5G39390.1          SALEYLHVYCHDPVAHCDIKPSNVLLDDDLTAHVSDFGLARLLYNFDEKTFLNQFSSAG-
AT2G24130.1          EGIAYLHHYSPVKVVHCDLKPSNILLDDEMTALVTDFGISRLVQGVEETVSTDDSVSFGS
AT5G46330.1          SGIDYLHSGYGFPIVHCDLKPANILLDSDRVAHVSDFGTARILGFREDGSTTASTSAFE- 1038
                     . : ***      :.***:**:*:**:.: .* *:*** ::::    :       .   :

AT3G47090.1          ----VRGTIGYAAPEYGMGGQPSIHGDVYSFGVLVLEMFTGKRPT--NELFGGNFTLNSY
AT3G47580.1          ----VRGTIGYAAPEYGMGGQPSIHGDVYSFGVLLLEMFTGKRPT--DELFGGNLTLHSY
AT3G47570.1          ----VRGTIGYAAPEYGVGGQPSINGDVYSFGILLLEMFTGKRPT--NELFGGNFTLNSY
AT3G47110.1          ----VRGTIGYAAPEYGMGGHPSIMGDVYSFGIVLLEIFTGKRPT--NKLFVDGLTLHSF
AT5G20480.1          ----VRGTIGYAAPEYGMGGQPSIQGDVYSFGILLLEMFSGKKPT--DESFAGDYNLHSY
AT5G39390.1          ----VRGTIGYAAPEYGMGSKPSIQGDVYSFGVLLLEMFTGKKPT--DNSFGGGYNLHGY
AT2G24130.1          TDGLLCGSVGYIAPEYGMGKRASTHGDVYSFGVLLLEIVSGRRPT--DVLVNEGSSLHEF
AT5G46330.1          ------GTIGYLAPEFAYMRKVTTKADVFSFGIIMMELMTKQRPTSLNDEDSQDMTLRQL 1092
                           *::** ***:.    : :  .**:***:::*:.: ::**  :      . .*.

AT3G47090.1          TKAALPER-VLDIADKSILHSGLRVGFPVLECLK-----GILDVGLRCCEESPLNRLATS
AT3G47580.1          TKLALPEK-VFEIADKAILHIGLRVGFRTAECLT-----LVLEVGLRCCEEYPTNRLATS
AT3G47570.1          TKSALPER-ILDIVDESILHIGLRVGFPVVECLT-----MVFEVGLRCCEESPMNRLATS
AT3G47110.1          TKSALQKRQALDITDETILRGAYAQHFNMVECLT-----LVFRVGVSCSEESPVNRISMA
AT5G20480.1          TKSILSGC------------TSSGGSNAIDEGLR-----LVLQVGIKCSEEYPRDRMRTD
AT5G39390.1          TKSVLSCS------------TSRGGRTMVDEWLR-----LVLEVGIKCSEEYPRDRMGMA
AT2G24130.1          MKSHYPDS-LEGIIEQALSRWKPQGKPEKCEKLWREVILEMIELGLVCTQYNPSTRPDML
AT5G46330.1          VEKSIGNGRKGMVRVLDMELGDSIVSLKQEEAIED-----FLKLCLFCTSSRPEDRPDMN 1147
                      :                             * :       .: : : * .   *   *

AT3G47090.1          EAAKELISIRERFFKTRRTARR----------------------
AT3G47580.1          EVAKELISIRERFFKTRRTPRR----------------------
AT3G47570.1          IVVKELISIRERFFKASRTTWR----------------------
AT3G47110.1          EAISKLVSIRESFFRDEET-------------------------
AT5G20480.1          EAVRELISIRSKFFSSKTTITESPRDAPQSSPQEWMLNTDMHTM
AT5G39390.1          EAVRELVSIKSKFFTSSR--------------------------
AT2G24130.1          DVAHEMGRLKEYLFACPSLLHFSSQETQGEASS-----------
AT5G46330.1          EILTHLMKLRGKANSFREDRNEDREV (1173 = end of FLS2)
                         .:  ::
```

Supplemental Figure 6. Lack of conservation of FLS2 Ser-938 in related non-RD kinases. Sequence alignment of kinase domains of non-RD kinases from LRR-RLK subfamily XII in Arabidopsis (Shiu and Bleecker, 2001). Red arrow indicates phosphorylation site Ser-938 of FLS2, which resides in a 10 amino acid region that aligns only poorly with the closest related Arabidopsis kinases. Black arrow indicates the conserved D997 in kinase active site.

Shiu SH, and Bleecker AB, 2001. Receptor-like kinases from Arabidopsis form a monophyletic gene family related to animal receptor kinases. Proc Natl Acad Sci. 98: 10763-10768.
