# Supplementary material for: Mutations in FLS2 Ser-938 Dissect Signaling Activation in FLS2-Mediated Arabidopsis Immunity
Source: PLoS Pathog. 2013 Apr 18;9(4):e1003313. doi: 10.1371/journal.ppat.1003313 (PMC3630090; doi:10.1371/journal.ppat.1003313)
Supplement: Figure S7 — Functional test of the response to elf18 peptide mediated by EFR Ser-777, Ser-778, and Ser-781 mutants. (PDF) [file ppat.1003313.s007.pdf]

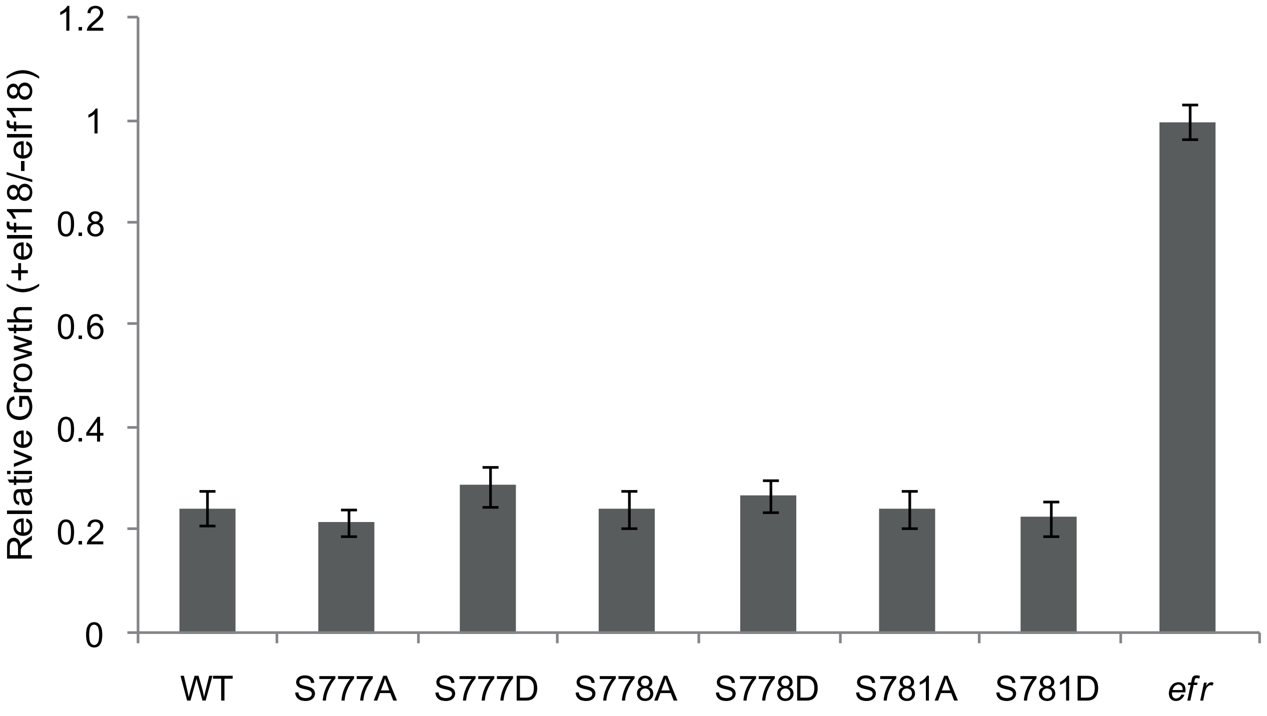

Supplemental Figure 7. Functional test of the response to elf18 peptide mediated by EFR Ser-777, Ser-778, and Ser-781 mutants. Three serines sites of EFR predicted to be in the protein region analogous to Ser938 of FLS2 were mutated to A or D and the resulting *EFR* alleles or wild-type (WT) *EFR*, all under control of the *EFR* native promoter were transformed into Col-0 *efr* mutant plants. T1 transgenic seedlings were used for seedling growth inhibition assay to test their responses to elf18. Relative growth was measured as the ratio of fresh weight of seedlings grown in the presence of 100 nM elf18 compared to the fresh weight of seedlings grown in the absence of elf18. Mean ±SE are shown (n=6).
