# Supplementary material for: Mutations in FLS2 Ser-938 Dissect Signaling Activation in FLS2-Mediated Arabidopsis Immunity
Source: PLoS Pathog. 2013 Apr 18;9(4):e1003313. doi: 10.1371/journal.ppat.1003313 (PMC3630090; doi:10.1371/journal.ppat.1003313)
Supplement: Figure S8 — Ser-938 may be important for phosphorylation of FLS2 in vitro and in vivo. (PDF) [file ppat.1003313.s008.pdf]

Supplemental Figure 8

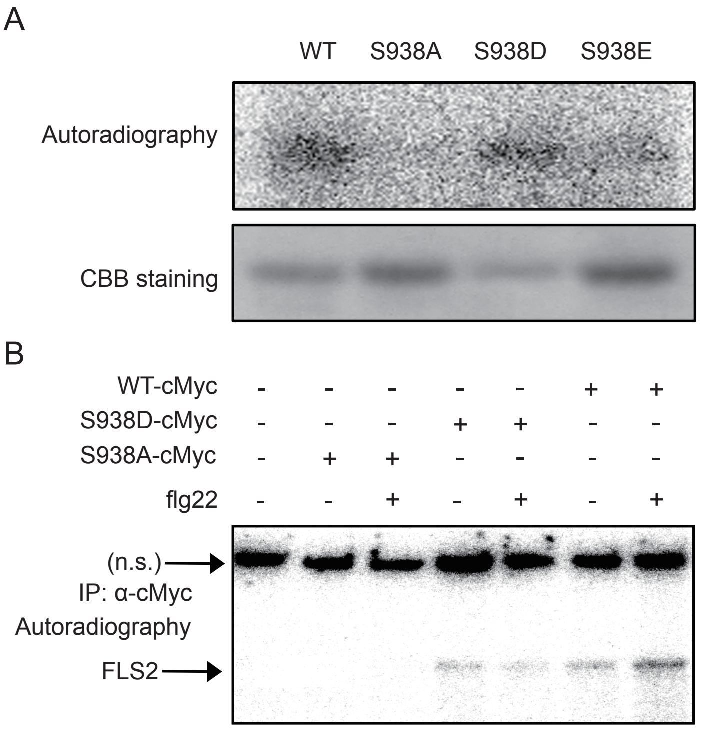

Supplemental Figure 8. Ser-938 may be important for phosphorylation of FLS2 in vitro and in vivo.

A. In vitro autophosphorylation activity of purified FLS2 kinase domains expressed from *E. coli* (GST tag removed during purification). Samples were separated by SDS-PAGE and then subjected to autoradiographic detection of ^{32}P. Lower panel: same samples, stained with Coomassie brilliant blue. Previous studies have also reported that FLS2 protein kinase activity is weak and/or difficult to detect in vitro [14,43,44]. In our experiments, FLS2WT had very low autophosphorylation activity that was only detected in some experiments, using high sensitivity settings on the phosphorimager. However, in experiments where autophosphorylation activity of FLS2WT was detected, FLS2S938A lacked activity, suggesting that Ser-938 may be required for the autophosphorylation activity of FLS2. FLS2S938D and FLS2S938E apparently retained very weak autophosphorylation activity.

B. In vivo phosphorylation of FLS2WT, FLS2S938A, and FLS2S938D before (-) or 15 min. after (+) exposure to 1 µM flg22. Col-0 *fls2-101* protoplasts transformed with the indicated full-length FLS2-myc alleles or PEG-only negative control were incubated for 10 hours in media carrying ^{32}P inorganic phosphate prior to collection, immunoprecipitation using anti-cMyc antibody, separation by SDS-PAGE and autoradiographic detection of ^{32}P. Note that gel run was very long (lower full-length FLS2 band migrates at ~170 kDa); upper band next to interface with stacking gel migrates at an extremely high apparent M.W. and is non-specific (n.s.; also present in control with no myc tag). The observed phosphorylation of FLS2S938D indicates that Ser-938 is not the only phosphorylation site on FLS2. The absence of phosphorylation of FLS2S938A is a more tentative observation because, although similar overall protein amounts were loaded in all lanes, the presence of ^{32}P prevented use of x-ray film to confirm presence of FLS2S938A using anti-myc antibody. However, FLS2-S938D-myc and FLS2-WT-myc are visible in this experiment due to ^{32}P labeling, and in other experiments with FLS2S938A its levels were similar to those of FLS2S938D and FLS2WT (e.g., Fig. 5, Fig. 6, Supplemental Fig. 2 (which includes Endo H tests for normal ER processing), and unpublished experiments).
