# Supplementary material for: Mutations in FLS2 Ser-938 Dissect Signaling Activation in FLS2-Mediated Arabidopsis Immunity
Source: PLoS Pathog. 2013 Apr 18;9(4):e1003313. doi: 10.1371/journal.ppat.1003313 (PMC3630090; doi:10.1371/journal.ppat.1003313)
Supplement: Figure S9 — Second example (see also Figure 4) of transphosphorylation of FLS2 kinase domain by BIK1. (PDF) [file ppat.1003313.s009.pdf]

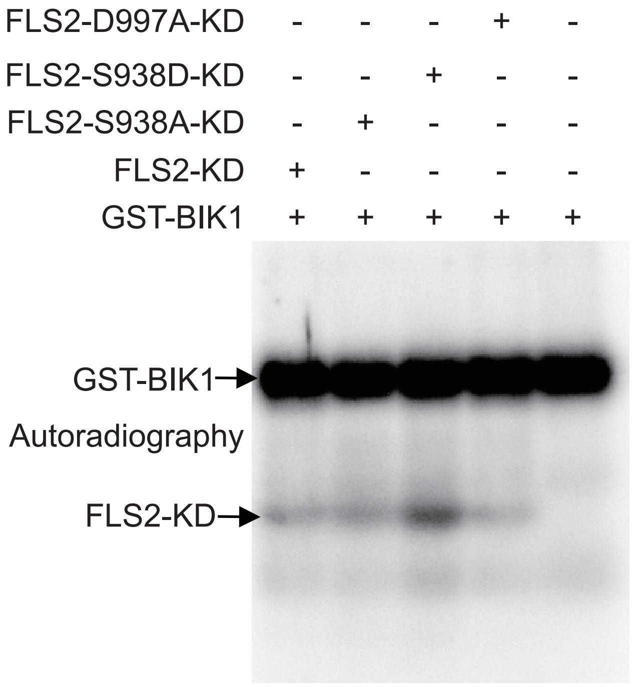

Supplemental Figure 9. Second example (see also Figure 4) of transphosphorylation of FLS2 kinase domain by BIK1. Kinase domains of FLS2 (wild-type or mutant as specified), and full-length wild-type BIK1 were purified from E. coli as GST fusion proteins; GST was cleaved from FLS2 proteins during purification. In vitro kinase assays carried out in the presence of [γ^{32}P]-ATP. Autoradiograph after SDS-PAGE.
