# Supplementary material for: Mutations in FLS2 Ser-938 Dissect Signaling Activation in FLS2-Mediated Arabidopsis Immunity
Source: PLoS Pathog. 2013 Apr 18;9(4):e1003313. doi: 10.1371/journal.ppat.1003313 (PMC3630090; doi:10.1371/journal.ppat.1003313)
Supplement: Table S1 — Primer sequences used in this study. (PDF) [file ppat.1003313.s010.pdf]

Name	Sequence (5'-3')
FLS2-S909A-F	GAATTCTCTGCAGAAGCAGACAAGTGGTTCT
FLS2-S909A-R	AGAACCACTTGTCTGCTTCTGCAGAGAATTC
FLS2-S909D-F	GAATTCTCTGCAGAAGACGACAAGTGGTTCTAC
FLS2-S909D-R	GTAGAACCACTTGTCGTCTTCTGCAGAGAATTC
FLS2-S909E-F	GAATTCTCTGCAGAAGAAGACAAGTGGTTCTAC
FLS2-S909E-R	GTAGAACCACTTGTCTTCTTCTGCAGAGAATTC
FLS2-S938A-F	GGGTTTGCGTGGGAAGCCGGCAAAACGAAAGCT
FLS2-S938A-R	AGCTTTCGTTTTGCCGGCTTCCCACGCAAACCC
FLS2-S938D-F	GGGTTTGCGTGGGAAGACGGCAAAACGAAAGCT
FLS2-S938D-R	AGCTTTCGTTTTGCCGTCTTCCCACGCAAACCC
FLS2-S938E-F	GGGTTTGCGTGGGAAGAAGGCAAAACGAAAGCT
FLS2-S938E-R	AGCTTTCGTTTTGCCTTCTTCCCACGCAAACCC
FLS2-S1084A-F	GTTGAATGATGAAGATGCACAAGACATGACTTTGC
FLS2-S1084A-R	GCAAAGTCATGTCTTGTGCATCTTCATCATTCAAC
FLS2-S1084D-F	GTTGAATGATGAAGATGACCAAGACATGACTTTGC
FLS2-S1084D-R	GCAAAGTCATGTCTTGGTCATCTTCATCATTCAAC
FLS2-S1084E-F	GTTGAATGATGAAGATGAACAAGACATGACTTTGC
FLS2-S1084E-R	GCAAAGTCATGTCTTGTTCATCTTCATCATTCAAC
BIK1-F	ATGGGTTCTTGCTTCAGTTCTCG
BIK1-R	CACAAGGTGCCTGCCAAAAGGTT
PBS1-F	ATGGGTTGTTTCTCGTGTTTTG
PBS1-R	CCCGGTACTGTTGCTCTCTG
PBL1-F	ATGGGTTCTTGTC TCAGTTC
PBL1-R	CAATCCAACGGTTTTTTTG
PBL2-F	ATGGGTAATT GTTTAGATTC
PBL2-R	TCTTACACGAGGAGATTGAG
FLS2T867D-F	GACGATTCATTCAACAGTGC
FLS2T867D-R	CTGTTGAATGAATCGTCTGCTTGCTCCAACTCTTTTG
FLS2-Kinase-F	CATGGATCCTGCAAGAAAAAAGAAAAAAAG
FLS2-R	AACTTCTCGATCCTCGTTACGATC
Prom-FLS2-F	CTGTTGAATGAATCGTCTGCTTGCTCCAACTCTTTTG

Supplemental Table 1. Primer sequences used in this study.
